# Primary Respiratory Chain Disease Causes Tissue-Specific Dysregulation of the Global Transcriptome and Nutrient-Sensing Signaling Network

**DOI:** 10.1371/journal.pone.0069282

**Published:** 2013-07-24

**Authors:** Zhe Zhang, Mai Tsukikawa, Min Peng, Erzsebet Polyak, Eiko Nakamaru-Ogiso, Julian Ostrovsky, Shana McCormack, Emily Place, Colleen Clarke, Gail Reiner, Elizabeth McCormick, Eric Rappaport, Richard Haas, Joseph A. Baur, Marni J. Falk

**Affiliations:** 1 Center for Biomedical Informatics, The Children’s Hospital of Philadelphia, Philadelphia, Pennsylvania, United States of America; 2 Division of Human Genetics, Department of Pediatrics, The Children’s Hospital of Philadelphia and Perelman School of Medicine, University of Pennsylvania, Philadelphia, Pennsylvania, United States of America; 3 Department of Genetics, Perelman School of Medicine, University of Pennsylvania, Philadelphia, Pennsylvania, United States of America; 4 Department of Biochemistry and Biophysics, University of Pennsylvania, Philadelphia, Pennsylvania, United States of America; 5 Division of Endocrinology, Department of Pediatrics, The Children’s Hospital of Philadelphia and Perelman School of Medicine, University of Pennsylvania, Philadelphia, Pennsylvania, United States of America; 6 Division of Child Development and Metabolic Disease, The Children’s Hospital of Philadelphia and Perelman School of Medicine, University of Pennsylvania, Philadelphia, Pennsylvania, United States of America; 7 Department of Pediatrics, University of California San Diego, San Diego, California, United States of America; 8 Nucleic Acid and Protein Core Facility, The Children’s Hospital of Philadelphia, Philadelphia, Pennsylvania, United States of America; 9 Department of Physiology, and Institute for Diabetes, Obesity, and Metabolism, Perelman School of Medicine, University of Pennsylvania, Philadelphia, Pennsylvania, United States of America; Texas A&M University, United States of America

## Abstract

Primary mitochondrial respiratory chain (RC) diseases are heterogeneous in etiology and manifestations but collectively impair cellular energy metabolism. Mechanism(s) by which RC dysfunction causes global cellular sequelae are poorly understood. To identify a common cellular response to RC disease, integrated gene, pathway, and systems biology analyses were performed in human primary RC disease skeletal muscle and fibroblast transcriptomes. Significant changes were evident in muscle across diverse RC complex and genetic etiologies that were consistent with prior reports in other primary RC disease models and involved dysregulation of genes involved in RNA processing, protein translation, transport, and degradation, and muscle structure. Global transcriptional and post-transcriptional dysregulation was also found to occur in a highly tissue-specific fashion. In particular, RC disease muscle had decreased transcription of cytosolic ribosomal proteins suggestive of reduced anabolic processes, increased transcription of mitochondrial ribosomal proteins, shorter 5′-UTRs that likely improve translational efficiency, and stabilization of 3′-UTRs containing AU-rich elements. RC disease fibroblasts showed a strikingly similar pattern of global transcriptome dysregulation in a reverse direction. In parallel with these transcriptional effects, RC disease dysregulated the integrated nutrient-sensing signaling network involving FOXO, PPAR, sirtuins, AMPK, and mTORC1, which collectively sense nutrient availability and regulate cellular growth. Altered activities of central nodes in the nutrient-sensing signaling network were validated by phosphokinase immunoblot analysis in RC inhibited cells. Remarkably, treating RC mutant fibroblasts with nicotinic acid to enhance sirtuin and PPAR activity also normalized mTORC1 and AMPK signaling, restored NADH/NAD^+^ redox balance, and improved cellular respiratory capacity. These data specifically highlight a common pathogenesis extending across different molecular and biochemical etiologies of individual RC disorders that involves global transcriptome modifications. We further identify the integrated nutrient-sensing signaling network as a common cellular response that mediates, and may be amenable to targeted therapies for, tissue-specific sequelae of primary mitochondrial RC disease.

## Introduction

Primary mitochondrial disease represents a heterogeneous group of genetic disorders that directly impair activity of the energy-generating respiratory chain (RC), with manifestations of severe and typically progressive multi-organ dysfunction that may present across the age spectrum. The mechanism(s) by which primary RC dysfunction causes such global cellular sequelae have not been well understood [1]. As a consequence, RC disease therapies have been largely focused on empiric supplements postulated to generically enhance residual mitochondrial oxidative phosphorylation capacity and reduce oxidative stress [2]. Unfortunately, these therapies remain largely ineffective.

Our prior investigations in animal models of primary mitochondrial disease have identified a consistent transcriptome response conserved from *C. elegans* to mice that involves significant dysregulation of central pathways involved in intermediary metabolism and transcriptional signaling [3], [4]. In particular, we found that the *PPAR* signaling pathway, which is involved in coordinating basic lipid metabolism, plays a central role in modulating hepatic and renal responses to primary RC dysfunction that results from a coenzyme Q biosynthetic deficiency in B6.*Pdss2^kd/kd^* mutant mice [5]. These findings suggest that a few master genes or central signaling pathways may modulate the transcriptional, translational, and/or post-translational cellular response to primary mitochondrial disease, and that this response may itself contribute to the pathogenesis of RC disease. Defining such central pathway alterations might therefore offer novel pharmacologic targets for treating the clinical sequelae of primary RC disease.

To identify a common cellular response to primary RC that might improve mechanistic understanding and lead to targeted therapies for human RC disease, we performed collective transcriptome profiling in skeletal muscle biopsy specimens and fibroblast cell lines (FCLs) of a diverse cohort of human mitochondrial disease subjects relative to controls. Systems biology investigations of common cellular responses to primary RC disease revealed a collective pattern of transcriptional, post-transcriptional and translational dysregulation that occurred in a highly tissue-specific fashion. In particular, a common transcriptional and post-transcriptional response to primary RC dysfunction entails reduction of cytosolic ribosomes, increase in mitochondrial ribosomes, decrease in 5′-UTR transcription to improve translational efficiency, and prolongation of 3′-UTR length to stabilize mRNA transcripts. In addition, these data highlight a central role of an integrated nutrient-sensing signaling network in the cellular response to primary RC disease, major components of which include FOXO, AMPK, PPAR, and sirtuins that are well-known cellular sensors of nutrient availability, as well as mTORC1 that is a key switch regulating cellular proliferation and growth. Altered activities of central nodes in the integrated nutrient-sensing signaling network were validated by phosphokinase immunoblot analyses in human FCLs and podocytes treated with RC inhibitors. Remarkably, treating RC complex I mutant fibroblasts with nicotinic acid, a known PPAR and sirtuin activator, also normalized mTORC1 and AMPK activities, restored NADH/NAD^+^ redox balance, and improved cellular respiratory capacity. These data are the first to implicate the integrated nutrient-sensing signaling network as a common cellular response mediating the sequelae of primary mitochondrial disease, which highlights potentially novel therapeutic targets to improve the manifestations of primary human RC disease.

## Materials and Methods

### Ethics Statement

All studies were completed following subject verbal and written informed consent from adult patients, and verbal and written informed consent from parents of pediatric patients together with child assent when appropriate, per Children’s Hospital of Philadelphia Institutional Review Board (IRB) approved study #08-6177 (M.J.F., PI).

### Subject and Tissue Sample Description

**Figure 1:**
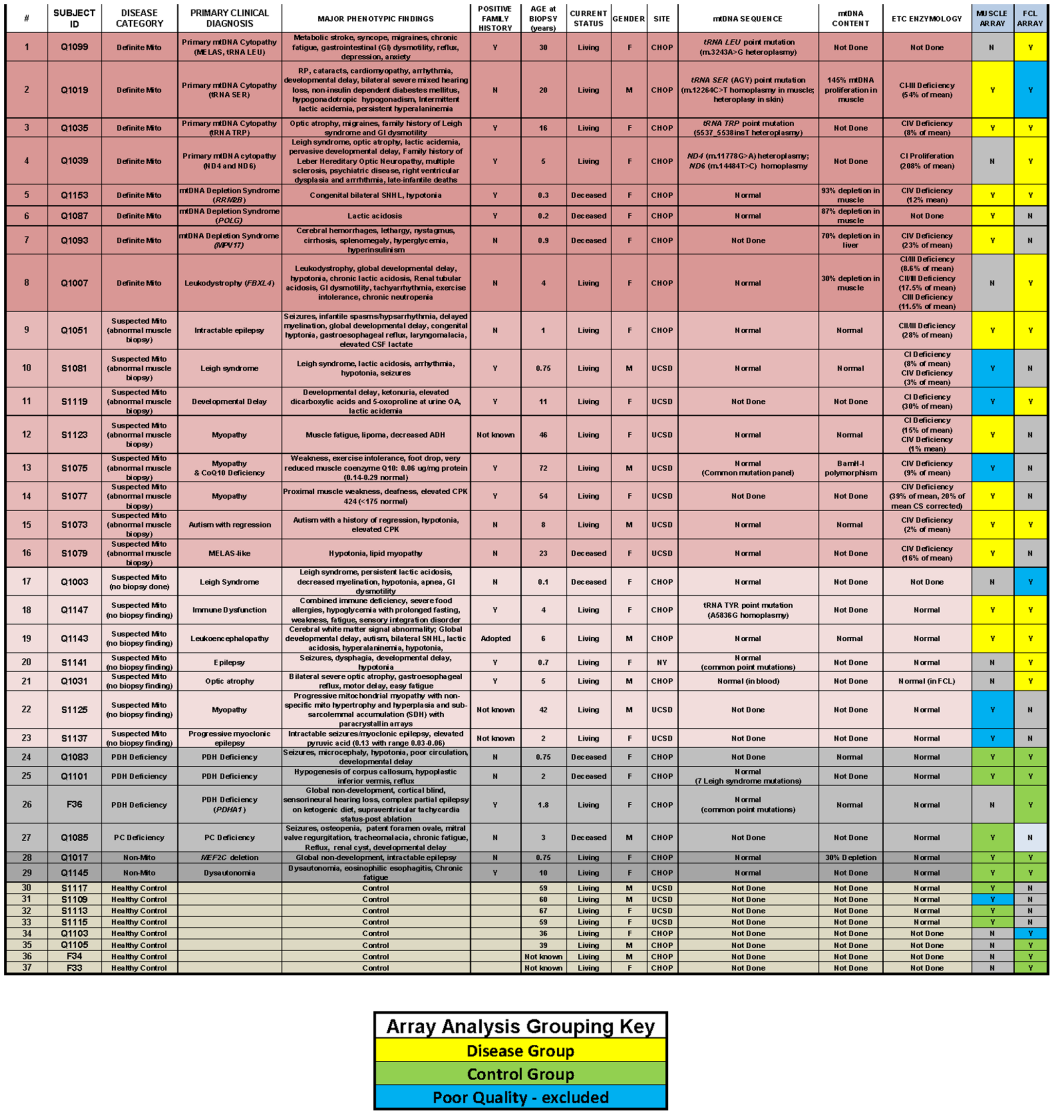
Detailed description of individual subjects’ clinical phenotypes, genetic and metabolic testing results, and tissue origins of RNA samples used for microarray analysis. Row highlights indicate disease groupings as “definite” primary mitochondrial RC disease (dark pink, known genetic etiology), “suspected” primary mitochondrial RC disease (mid-pink or pale-pink, as respectively based on biochemistry abnormal or normal/not done), pyruvate metabolism defect (light gray), non-mitochondrial other disease with normal muscle electron transport chain activity analysis (dark gray), or healthy control (white). The key indicates which muscle and FCL microarray datasets, as indicated in the last two columns, respectively, were ultimately selected for downstream analyses in either control (light green) or mitochondrial disease (yellow) cohorts, as well as samples not selected for analysis due to poor microarray data quality (blue). Additional details have been previously reported on subjects and/or their family members for subjects 1019 [79], 1035 [80], and 1039 [81].

Residual skeletal muscle biopsy specimens were obtained from Clinical Pathology at The Children’s Hospital of Philadelphia or University of California San Diego following completion of all clinical diagnostic assays with the express participant consent of all living participants and/or families, or from decedents following IRB approval. Clinical and biochemical characteristics of all subjects enrolled in this study on whom microarray datasets were generated are detailed in **Fig. 1**, with only samples that generated high quality transcriptome data ultimately included in bioinformatic analyses.

### Fibroblast Cell Line Culture

Fibroblast cell lines (FCLs) were obtained from prior skin biopsies when available and/or established in the Clinical CytoGenomics Laboratory from skin biopsies performed in Mitochondrial-Genetics Diagnostic Clinic at The Children’s Hospital of Philadelphia (M.J.F.), following informed consent. FCLs were grown in Dulbecco’s modified Eagle’s medium (DMEM, Gibco) containing 1 g/L glucose and supplemented with 20% FBS (Gibco), 1 mMol sodium pyruvate (CellGro), 2 mMol L-glutamine, and 50 ug/mL uridine (Calbiochem). FCLs for Western analysis were grown to 80–100% confluence in T75 flasks at 37°C before 24 hour treatment with varying concentrations of oligomycin from 0.25 uMol to 5 uMol, 2 mMol AICAR, or 100 nMol rapamycin. Cells were washed with Hank’s buffered salt solution (HBSS), trypsinized, flash frozen in conical 15 ml tubes, and cell pellets were lysed for 30 minutes on ice in 200 µl cold RIPA Buffer (Cell Signaling Technology) supplemented with phosphatase and protease inhibitors (Sigma-Aldrich). Samples were centrifuged at 10,000×*g* for 5 minutes at 4°C and supernatants were then transferred to fresh tubes. Protein concentration was determined by DC Protein Assay (Bio-Rad). Protein extract samples were treated with Laemmli Sample Buffer (Bio-Rad) with β-mercaptoethanol at 95°C for 4 minutes for immunoblot analyses.

### Total RNA Extraction

Total RNA was extracted from frozen FCL pellets per standard protocol by RNeasy Mini kit (Qiagen), after cells were placed in kit lysis buffer and processed by a QIAshredder homogenizer to increase RNA yield. Total RNA was extracted from liquid nitrogen flash-frozen skeletal muscle tissues by Norgen RNA/DNA/Protein purification kit (Norgen Biotek Corporation, Ontario, Canada), with slight modification. 10–20 mg of frozen muscle tissue was transferred into a plastic tube with tight fitting pestle and 300 ul of kit lysis buffer, homogenized, and mixed by vortexing after 600 ul of RNAse free water was added. Lysates were incubated with intermittent vortexing at 55°C for 15 minutes with 20 ul Proteinase K. Following centrifugation for 10 minutes, supernatants were mixed with equal volumes of 70% alcohol and RNA extraction was completed per published protocol (Norgen protocol booklet step 1.B.h). RNA was analyzed by NanoDrop 1000 spectrophotometer and Agilent 2100 Bioanalyzer in the Nucleic Acid and Protein Core Facility at The Children’s Hospital of Philadelphia, with RNA integrity number (RIN) >8 acceptable (maximum RIN is 10) for microarray and real-time qPCR analyses.

### Exon Microarray Data Processing

All microarray data processing and statistical analyses were performed in the R environment. Probe sequences of the Human Exon 1.0 ST Array (Affymetrix, Inc.) were aligned to Hg19 reference genome. Perfectly aligned probes not overlapping to any dbSNP entries were grouped into probesets based on RefSeq gene annotation. Probesets mapped to the same NCBI gene were further grouped to obtain gene-level data. Probe-level data were normalized by the Lowess method and summarized into probeset-level measurements of transcript abundance with the Li-Wong algorithm. The same procedure was applied to probeset-level data to obtain gene-level measurements. Differential expression analyses were performed with SAM (Significance Analysis of Microarrays) method. Three functional annotation tools were used. Gene set enrichment analysis (GSEA, http://www.broadinstitute.org/gsea) was applied to evaluate for concordance in differential expression among genes of the same KEGG pathway or Gene Ontology term in RC disease. The Database for Annotation, Visualization and Integrated Discovery (DAVID, http://david.abcc.ncifcrf.gov) tool was used to look for gene sets over-represented in groups of differentially expressed genes (DEGs), such as genes up-regulated in both RC disease muscle and RC disease FCLs. Ingenuity pathway analysis (IPA, http://www.ingenuity.com/products/ipa) was utilized to construct spontaneous gene networks from significantly DEGs. Details of data processing and individual bioinformatics analyses are described in **File S1**. All microarray data was deposited in the public NCBI database (GEO series #GSE42986).

### Expression Microarray Cohort Bioinformatics Analysis Dataset Details

We classified subjects for this study as having either “definite” or “suspected” mitochondrial diseases, where all had convincing clinical and/or biochemical presentations for primary mitochondrial disease but only in the former were clearly pathogenic gene mutations known. Bioinformatics analyses were performed to compare skeletal muscle expression array data from the 12 individuals with definite (Q1019; Q1035; S1073; S1077; S1079; Q1087; Q1093; S1123; Q1153) or suspected (Q1051, Q1143, Q1147) mitochondrial RC disease to 8 individuals with normal RC function as quantified by electron transport chain (ETC) enzyme activity analyses (Q1017; Q1083; Q1085; Q1101; S1113; S1115; S1117; Q1145). Similarly, bioinformatics analyses were performed to compare FCL expression array data from 12 individuals with definite (Q1007; Q1035; Q1039; S1073; Q1099, S1119; Q1153;) or suspected (Q1031, Q1051; S1141, Q1143, Q1147) mitochondrial RC disease to 8 individuals who had non-mitochondrial respiratory chain diseases and/or were healthy controls (Q1017; Q1083; Q1101; Q1105; Q1145; F33; F36; F34). Both skeletal muscle and FCL expression arrays were studied from 10 subjects, including 3 individuals with definite RC disease (Q1035, S1073, Q1153,), 3 individuals with suspected RC disease (Q1051, Q1143, Q1147), and 4 individuals grouped as controls for this study since they had known genetic disorders that do not involve RC dysfunction (Q1017, Q1083, Q1101, Q1145). Clinical information on these subjects including disease categorization, primary clinical diagnosis, major phenotypic findings, family history, age at biopsy, gender, mtDNA genome sequence results, mtDNA content analysis results, and ETC enzyme activity analysis results are detailed in **Fig. 1**. Due to poor microarray data quality, array data was excluded from downstream analyses in 6 muscle samples (S1075, S1081, S1109, S1119, S1125, S1137) and 3 FCL samples (Q1003, Q1019, Q1103). Thus, muscle data was ultimately analyzed from a total of 12 individuals with mitochondrial disease were grouped into the ‘RC disease cohort’ for subsequent analyses, which included both genders (9 F, 3 M), a wide age range (infancy through 54 years) and variable clinical disease severity (4 deceased, 8 living) relative to a matched “control cohort” that included both genders (6 F, 2 M), a similarly wide age range (infancy to 67 years), and similar spectrum of disease severity (3 deceased, 5 living). FCL datasets of high quality were also ultimately studied on a total of 12 RC disease subjects and 8 control subjects, of which 6 and 4 subjects, respectively, also had muscle expression datasets analyzed in this study). The FCL “RC disease cohort” thus included both genders (9 F, 3 M), wide age range age (infancy to 30 years), and variable clinical disease severity (1 now-deceased and 11 living subjects) relative to a matched FCL control cohort was similarly matched by gender (6 F, 2 M), age range (infancy to 39 years), and clinical severity (2 now-deceased and 6 living subjects).

### Phosphokinase Immunoblot Analyses

100 µg of protein was separated by SDS-PAGE on a 4–15% Tris-Glycine gradient gel (Bio-Rad) at 30 mA x1 hour, transferred to nitrocellulose membranes at 350 mA ×1.5 hours, and incubated in Odyssey blocking buffer ×1 hour. Phospho-proteins were labeled with rabbit anti-phospho antibodies at 1∶2,000 dilutions at 4°C for 1 hour or overnight. Membranes were incubated with Odyssey IRDye Goat anti-Rabbit or anti-Mouse Secondary Antibody at 1∶10,000 dilutions for 30 minutes. Protein bands were visualized by the Odyssey Infrared Imaging System (LI-COR Biosciences). Specific antibodies used for immunoblot analyses were Phospho(P)-AMPKα (2535), AMPKα (2532), P-S6 ribosomal protein (2215), and S6 ribosomal protein (2217) (Cell Signaling Technology). β-tubulin (Abcam) and/or β-actin (GenScript) were used as loading controls. Biologic triplicate analyses were performed per condition.

### Sample Preparation for HPLC Analyses of NAD^+^ and NADH

Harvested cells were rinsed with Hank’s balanced salt solution twice and centrifuged at 2,150×g for 5 minutes. The cell pellet was resuspended with argon-bubbled 20 mMol Tris-HCl PH 7.4, and divided into two tubes: one for analysis of the oxidized dinucleotides including NAD^+^ and the other for analysis of the reduced dinucleotides including NADH. For determination of NAD^+^, cell suspension was extracted with 4 volumes of argon-bubbled ice-cold 1.2 Mol perchloric acid (PCA) containing 20 mMol EDTA and 0.15% of sodium metabisulfite. After vortexing, the suspension was placed on ice for 15 minutes and then centrifuged at 16,000×g for 10 minutes. The supernatant was neutralized with 1 Mol potassium carbonate and centrifuged to remove insoluble material. The pellet from the PCA extraction was used for protein estimation. For determination of NADH, cell suspension was extracted with 4 volumes of ice-cold acetonitrile: 50 mMol ammonium acetate (1∶1 v/v) containing 50 mMol NaOH. The suspension was vortexed, placed on ice for 15 minutes, and then centrifuged at 16,000×g for 10 minutes to remove insoluble material. The supernatant was transferred to a spin column (50 K MWCO) and subsequently centrifuged at 16,000×g rpm for 90 minutes to remove macromolecules in the sample. The eluate was transferred to a new tube and was placed on ice under a stream of argon for 10 to 15 minutes to remove acetonitrile. Samples were stored at −80°C and subjected to HPLC analysis.

### HPLC Conditions

Separation of the oxidized and reduced dinucleotides was carried out on a C18 column (5 mm, 4.6×250 mm) preceded by a guard column at 35°C. Flow rate was set at 0.5 mL/min. The mobile phase was initially 100% of mobile phase A (0.1 Mol potassium phosphate buffer, pH 6.0, containing 3.75% methanol). The methanol was lineally increased with mobile phase B (0.1 Mol potassium phosphate buffer, pH 6.0, containing 30% methanol) increasing to 50% over 15 minutes. The column was washed after each separation by increasing mobile phase B to 100% for 5 minutes. UV absorbance was monitored at 260 and 340 nm with Shimadzu SPD-M20A. Pertinent peak areas were integrated by the LabSolution software from Shimadzu, and quantified using standard curves. Statistical analyses were performed between groups using Student’s 2-tailed *t*-test.

### High Resolution Respirometry in Intact Cells using Oxygraph-2K (Oroboros)

Cells were cultured in tissue culture flasks until reaching 80% confluence and then prepared for respiratory capacity analysis by high resolution polarography using an Oxygraph-2K (Oroboros, Austria), as previously described [6]. Cells were removed from culture flasks with trypsinization and collected in fresh culture medium. Control (normal) and mitochondrial disease patient cell lines were simultaneously analyzed in two separate chambers in 2 ml volume containing 1 million cells in each chamber. Experiments were performed as previously described [7]. Respiration was measured at 37°C in culture medium (DMEM), where inhibitors for the different mitochondrial respiratory complexes were added to the cells in the following order: oligomycin (2 ug/ml) (Sigma) to inhibit complex V (to assess non-mitochondrial respiratory capacity or leak rate), carbonyl cyanide-p-trifluoromethoxyphenylhydrazone (FCCP) (Sigma) uncoupler with step-wise titration in 2.5 to 1.5 uMol increments (to assess maximal electron transport system respiratory capacity rate), rotenone (Sigma) in 0.5 uMol final concentration to inhibit complex I, and antimycin A (Sigma) in 2.5 uMol final concentration to inhibit complex III. Data was analyzed using DatLab4 (Oroboros, Austria) software. Statistical analyses were performed using ANOVA and paired t-tests to compare group means.

### High Resolution Respirometry in Permeabilized Cells using Oxygraph-2K (Oroboros)

Cells were cultured and prepared for high resolution respirometry as described above, except that after trypsinization cells were washed with Dulbecco’s phosphate buffered saline following one wash with respiration medium containing 0.5 mMol EGTA, 3 mMol MgCl_2_×6H_2_O, 20 mMol taurine, 10 mMol KH_2_PO_4_, 20 mM HEPES, 1 g/L BSA, 60 mMol potassium-lactobionate, 110 mMol mannitol, and 0.3 mMol DTT, pH = 7.1. Cells were permeabilized using digitonin according to published protocol [8], [9]. Substrates and inhibitors were added in the following order: 10 mMol glutamate, 5 mMol malate, 3 mMol ADP, 10 uMol cytochrome C, stepwise titration of FCCP uncoupler in 1.5 uMol to 2.5 uMol increment as needed, 0.5 uMol rotenone, 5 uMol antimycin A and 0.5 mMol TMPD/2 mMol ascorbate. Data was analyzed using DatLab4 (Oroboros, Austria) software. Statistical analyses were performed using ANOVA to compare group means.

## Results

### The Transcriptome is Significantly Altered in Primary RC Disease Skeletal Muscle

**Figure 2:**
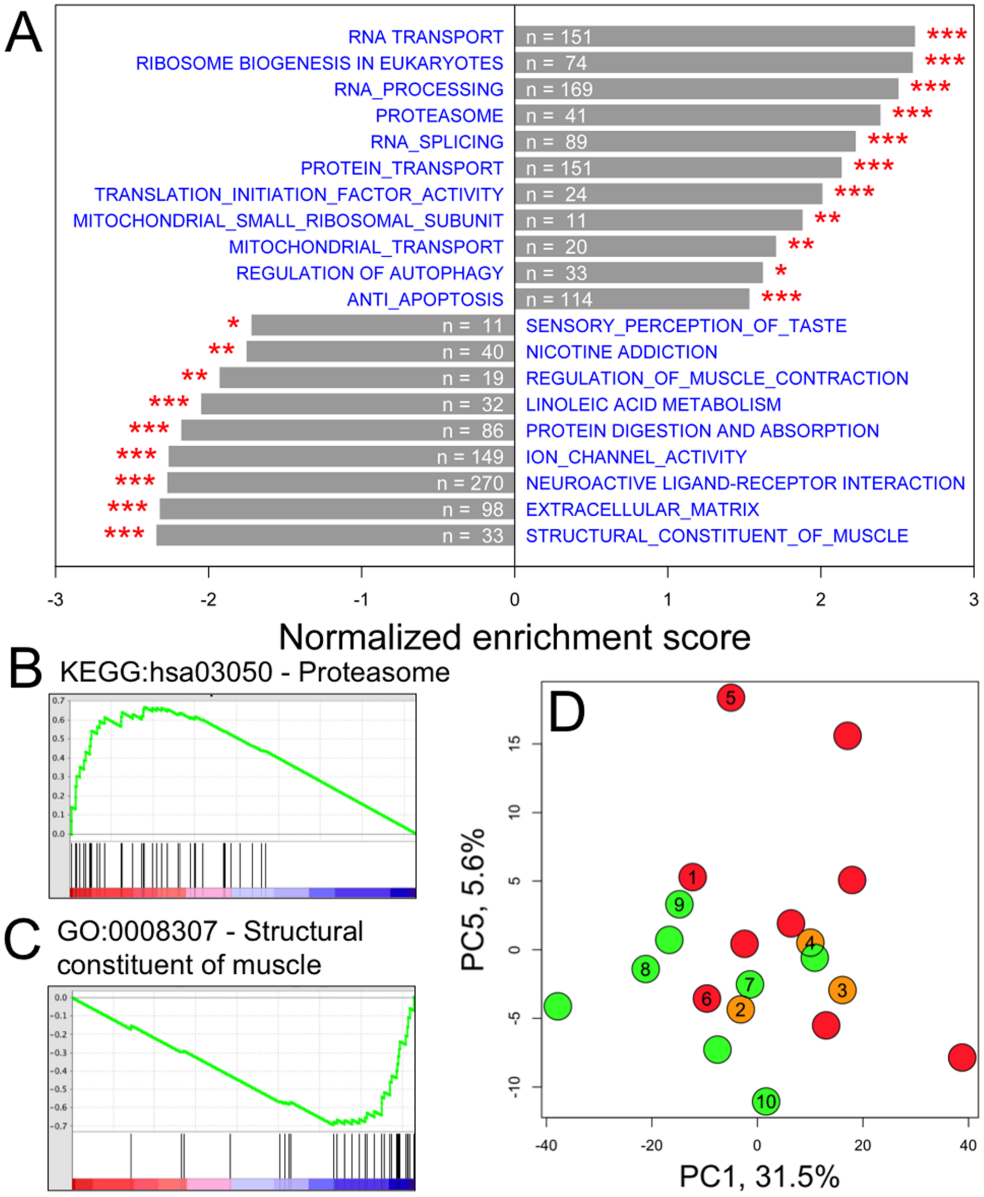
Differential gene expression in RC disease muscle. (**A**) Significantly differentially expressed gene sets identified by GSEA (See **File S2** for complete results). Positive and negative enrichment scores, respectively, indicate up- and down-regulation in RC disease. Numbers indicate counts of genes in each gene set that were measured by our experiment. * p<0.05; ** p<0.01; *** p<0.001. (**B–C**) Running enrichment scores of two significantly differentially expressed gene sets identified by GSEA. In each plot, the green and black lines, respectively, convey the running enrichment score and the genes categorized into the gene set as defined by KEGG pathway or GO gene ontology. The colored panel at the bottom indicates the level of differential expression from the most up-regulated (red) to most down-regulated (blue) in RC disease. (**B**) Proteasome pathway genes were generally up-regulated in RC disease muscle. (**C**) Structural muscle proteins were generally down-regulated in RC disease. (**D**) Unsupervised clustering via principal component analysis (PCA) identified distinctive transcriptome groupings among muscle from subjects categorized as color-detailed and defined in **Fig. 1** based on having “definite” RC disease (red), “suspected” RC disease with abnormal muscle RC biochemistry (dark pink), “suspected” RC disease with either no biopsy performed or no definitive biochemical abnormalities identified (light pink), pyruvate metabolism defect controls (light gray), ‘other disease” controls (dark gray), and healthy controls (brown). Numbered circles indicate subjects also studied in the fibroblast data set (**Fig. 3D**).

Probe-level microarray data were summarized into 20,741 unique NCBI Entrez genes prior to statistical comparison of transcriptomes (**File S1**). We first applied GSEA to compare differential gene expression between eight healthy controls and ten subjects with definite genetic RC disease etiologies and/or strong biochemical evidence of RC dysfunction (<30% of control mean) in the same skeletal muscle sample in which transcriptome analysis was performed (**Fig. 1**). GSEA revealed a number of gene pathways and categories that were significantly modified on average across all RC disease subjects’ muscle (**Fig. 2A**, full list is provided in **File S2**). For example, genes in the proteasome pathway were generally upregulated (**Fig. 2B**), while genes encoding structural muscle components were generally down-regulated (**Fig. 2C**). Unsupervised clustering of samples using principal components analysis (PCA) showed that subjects with “suspected” mitochondrial diseases who had either clinical and/or biochemical evidence of RC disease but no known underlying genetic etiology displayed gene expression patterns that were indistinguishable from the “definite” RC disease subjects and clearly different from the control subjects (**Fig. 2D**). Therefore, we grouped together all 12 RC disease subjects for further skeletal muscle transcriptome comparisons made relative to 8 controls with normal RC function. Overall, we identified 4,367 DEGs with p value less than 0.05 and an estimated 12.1% false discovery rate (FDR). 2,016 of these genes were upregulated and 2,351 of these genes were downregulated in RC disease. *RHEB*, a mediator of the nutrient-sensing mTORC1 pathway, was the most significantly changed gene in RC disease muscle (increased by 41%, p = 6.1E^−5^, SAM test).

To test the extent to which transcriptome changes in our heterogeneous pool of RC disease subjects’ muscle reflected more homogeneous subgroups of RC disease, we re-analyzed a public microarray data set generated by Crimi et al. [10] that investigated gene expression changes in skeletal muscle biopsies of mitochondrial encephalomyopathy (MEM) subjects organized into 3 subgroups including mitochondrial encephalopathy lactic acidosis and stroke (MELAS), progressive external ophthalmoplegia (PEO), and common 4977 base pair mitochondrial DNA (mtDNA) deletion subjects. We identified 35 genes that were commonly upregulated in all 3 MEM subgroups (**Fig. S1 in File S3**). This set of 35 genes was also significantly upregulated in our RC disease muscle group relative to controls by an average of 8.6% (p = 7.0E^−5^). Six genes (*DNAJC15*, *ESYT1*, *EXOC2*, *PGRMC2*, *SPCS3*, and *UBXN2B*) were significantly upregulated in all MEM subgroups as well as in our RC disease muscle, all of which encode membrane proteins that relate to protein metabolism or transport (**Table S1 in File S3**). For example, *EXOC2* encodes an exocyst complex component and the UBXN2B protein contains a ubiquitin domain.

### Primary Mitochondrial RC Disease Inversely Modifies Muscle and Fibroblast Transcriptomes

**Figure 3:**
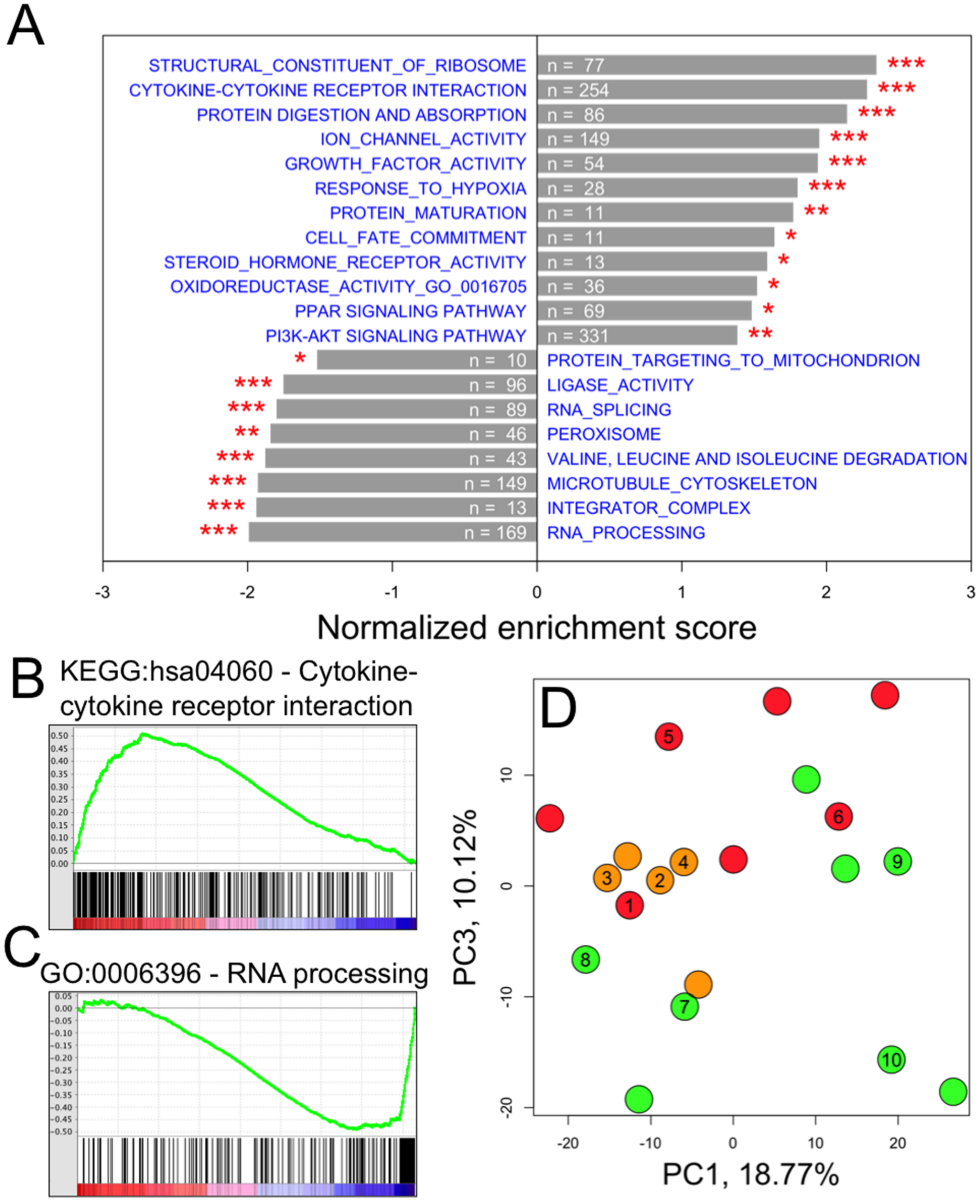
Differential gene expression in RC disease FCL. (**A–D**) Differential expression of the same GSEA-defined pathways are shown for RC disease versus control FCLs as identified in skeletal muscle in **Fig. 2** (See **File S2** for complete results). Positive and negative enrichment scores, respectively, indicate up- and down-regulation in RC disease. Numbers indicate counts of genes in each gene set that were measured by our experiment. * p<0.05; ** p<0.01; *** p<0.001. (**B**) The “Cytokine-cytokine receptor interaction” gene-set was identified in GSEA as among the most up-regulated gene sets in RC disease FCLs. (**C**) RNA processing pathway genes were generally down-regulated in RC disease FCLs. (**D**) Unsupervised clustering via principal component analysis (PCA) identified distinctive transcriptome groupings among FCLs from subjects categorized as color-detailed and defined in **Fig. 1**, where numbered circles indicate subjects also studied in the muscle data set (**Fig. 2D**).

We performed a second microarray experiment using 20 fibroblast cell line (FCL) samples from twelve RC disease subjects and eight controls, where 10 samples were from the same subjects studied in the skeletal muscle data set. Performing the same analyses as described for the muscle dataset, we first applied GSEA to compare 8 controls and 8 subjects with definite genetic RC disease etiologies and/or strong biochemical evidence of RC dysfunction (<30% of control mean) to identify gene sets concordantly modified in RC disease FCLs (**Fig. 3A** and **File S2**). For example, genes of the ‘cytokine-cytokine receptor interaction’ cluster were generally upregulated in RC disease FCLs (**Fig. 3B**), while genes involved in RNA processing were on average significantly downregulated (**Fig. 3C**). PCA analysis again showed that FCL data sets of the subjects with “suspected” mitochondrial diseases who had either clinical and/or biochemical evidence of RC disease but no known underlying genetic etiology had an overall transcriptome pattern more similar to the “definite” RC disease subjects (who had confirmed genetic etiology) than to the controls (**Fig. 3D**). After combining the “definite” and “suspected” subjects’ FCLs into a common “RC disease” group for comparison to control FCLs, 3,981 DEGs in RC disease FCLs were identified (p<0.05, FDR = 0.16). Among these, *RHEB* transcription was again found to be significantly changed (p = 0.016, SAM test), with reduced expression by 12% in RC disease FCLs.

**Figure 4:**
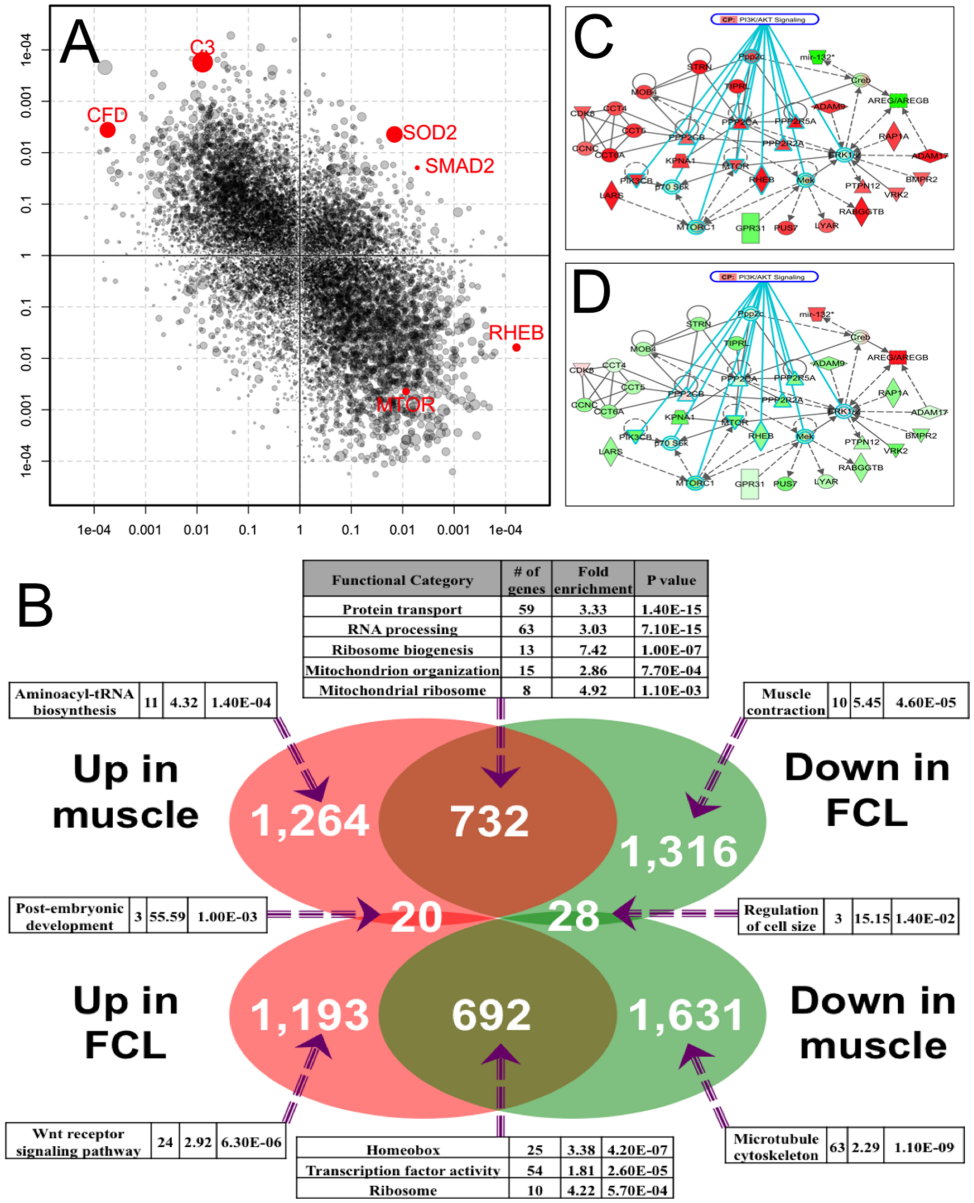
The global pattern of transcriptome changes in RC disease was reversed in muscle and FCL. (**A**) The global pattern of differential gene expression showed a highly significant negative correlation between RC disease muscle and FCLs (r = −0.49, p<10^−16^). The x- and y-axis units are signed p values, where dot size of individual genes is proportional to the combined fold-change of the two tissue types in RC disease relative to controls. (**B**) Genes were categorized based on their relative differential expression patterns in both muscle and FCL RC disease. Each subset was submitted to DAVID for over-representation analysis, where the most significant categories identified in DAVID are detailed for each of 8 unique gene subsets. (**C–D**) One of the spontaneously constructed IPA gene networks centered on MTOR. Red and green convey up- and down-regulation in RC disease, respectively. Twelve of the genes in this network have previously been associated to PI3K/AKT pathway signaling (blue lines). (**C**) Most genes in the MTOR IPA gene network showed significant up-regulation in RC disease muscle, with the exception of only a few downstream network members, such as *mir-132*. (**D**) Almost all genes identified in the IPA-centered MTOR network showed reversed expression change in RC disease FCL relative to controls as had been seen in muscle, including *mir-132* (**Fig. 4C**).

Indeed, the opposing change of differential gene expression in RC disease muscle and RC disease FCLs was found to be a global phenomenon (**Fig. 4A**). Among genes that were differentially expressed in both RC disease muscle and FCLs, 97% showed inverse directions of change in the two tissues. Such a global yet tissue-specific response to mitochondrial RC dysfunction is suggestive that one or several ‘master regulator(s)’ exist to sense primary RC dysfunction and mediate a cascade of downstream events to affect many genes and cellular processes.

While few genes were concordantly dysregulated, many of the 20 upregulated and 28 downregulated genes in RC disease that were common to both tissues did have apparent biologic relevance (**Table S2 in File S3**). For example, *SOD2* and *SMAD2* were among the genes significantly increased in both cell types. *SOD2* encodes the mitochondrial manganese superoxide dismutase that detoxifies mitochondrial superoxide [11]. *SMAD2* is a transcription factor (TF) that regulates cell proliferation in response to *TGF-beta* signaling [12]. Among the 28 concordantly downregulated genes, *LAMTOR2* activates the mTORC1 pathway [13] and *CITED2* co-activates PPAR-α dependent transcriptional regulation [14].

Over-representation of pre-defined gene categories in different DEG subsets was assessed using DAVID (**Fig. 4B**). The gene subset that was upregulated in muscle and downregulated in FCLs (“muscle-up/FCL-down”) was enriched for RNA processing, ribosome biogenesis, and protein transport, which is suggestive that post-transcriptional, translational, and post-translational regulation is involved in the common cellular response to RC disease. In contrast, the DEG subset that was downregulated in muscle and upregulated in FCLs (“muscle-down/FCL-up”) was enriched for HOX genes and growth factors, which may reflect poor muscle growth in RC disease. Additionally, genes regulating cell size were downregulated in RC disease in both tissue types, whereas genes involved in muscle contraction were downregulated only in RC disease muscle. The overall inverse DEG patterns might also be reflective, in part, of *in vivo* skeletal muscle metabolism, where nutrient availability is tightly controlled, as compared to *in vitro* FCL culture with unlimited nutrient availability. Nonetheless, functional characterization of DEGs was suggestive that RC disease affects a variety of basic cellular functions across both *in vivo* and *in vitro* settings in response to tissue-specific metabolic demands.

IPA spontaneously constructed several gene networks from DEGs of each tissue. One of the top networks centered on MTOR, which is a key element responding to cellular nutrient status and regulating protein translation. Most genes in this network were significantly upregulated in RC disease muscle, including genes involved in the PI3K/AKT signaling pathway (**Fig. 4C**). RC disease FCLs showed differential expression of this network in a direction that was completely reversed (**Fig. 4D**). A particularly noticeable gene at the downstream end of this network was *mir-132*, which is a microRNA that regulates cytokine production and is induced by nutritional stress [15]. While *mir-132* itself had significantly downregulated expression in RC disease muscle, the average expression of its target genes was significantly upregulated in RC disease, both of which were again reversed in RC disease FCLs (**Fig. S2 in File S3**). Interestingly, Tyynismaa et al. found that mitochondrial myopathy induced a starvation-like response in mice with multiple mtDNA deletions caused by an autosomal dominant mutation in the mitochondrial replicative helicase, Twinkle [16]. IPA performed on array data from that mitochondrial myopathy mouse model also identified the PI3K/AKT signaling pathway as among the most significantly altered, with a significant increase seen in the phosphorylated forms of AKT1 at amino acids 308 and 473 in the myopathic mice. Similarly, we found that the expression of the 16 genes most upregulated in the mitochondrial myopathy mice were also increased by an average of 7.5% in our human RC disease muscle dataset, with significant changes in 2 of these genes, *RC3H2* and *RIF1*.

Another differentially regulated gene of particular interest in RC disease was *LRPPRC*, which is known to interact with FOXO1 and PGC-1alpha proteins and is mutated in French-Canadian Leigh syndrome with RC complex IV deficiency [17]. Vishal et al. discovered that transcript expression of LRPPRC in human fibroblasts in which this gene had been knocked-down by RNA interference showed strong correlation with expression of mtDNA-encoded, but not nDNA-encoded, transcripts [18]. In our data sets, *LRPPRC* expression was significantly upregulated in RC disease muscle and showed marginally significant downregulation in RC disease FCLs. Using correlation analysis, we identified 53 genes that significantly and positively correlated to *LRPPRC* expression in both RC disease muscle and FCLs. Eighteen of these genes encode mitochondrial proteins, including 5 mitochondrial ribosomal proteins and 2 mitochondrial translational initiation factors. Based on these findings, we postulate that *LRPPRC* may play a role in the dysregulation of mitochondrial protein synthesis and transport that occurs in primary RC disease.

### Target Gene Co-regulation Identifies Specific TFs Whose Activity is Altered in Mitochondrial RC Disease

**Figure 5:**
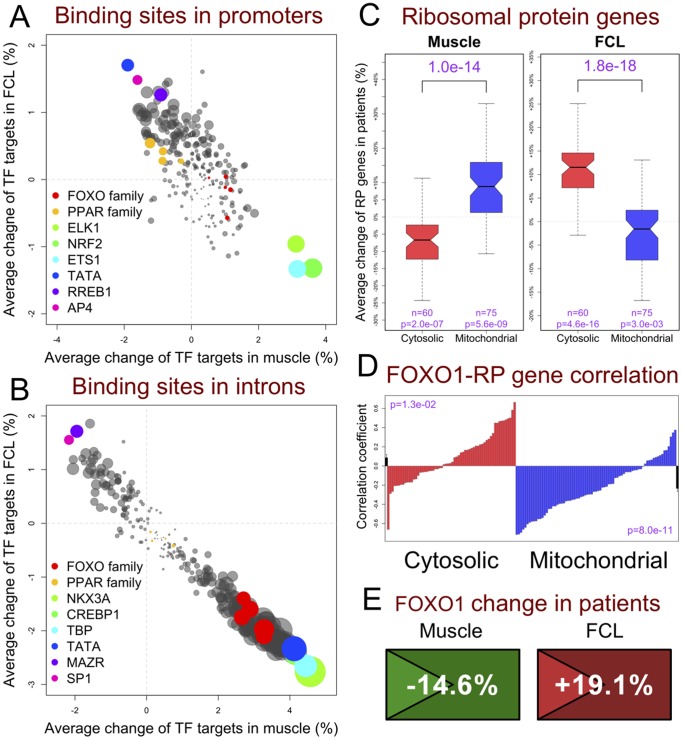
Co-regulation of gene groups by upstream regulators. (**A**) **TF activity analysis of genes with promoter binding sites.** Average change of genes whose [−1 kb, 1 kb] promoter regions containing sites matching the position weight matrix (PWM) of 258 transcription factors (TFs) indicated that many TFs had modified activity in primary RC disease. Each dot represents a known TF PWM and its size corresponds to the combined p value in muscle and FCLs. (**B**) **TF activity analysis of genes with intronic binding sites.** The same analysis as in (A) was performed on genes whose introns contained sites matching the PWMs. (**C**) **Inverse expression of cytosolic and mitochondrial ribosomal genes in RC disease.** Overall differential expression of cytosolic and mitochondrial RP genes in RC disease had opposite patterns in muscle and FCLs. This analysis included genes encoding ribosome subunits, but not RP pseudogenes and genes regulating ribosome biogenesis, such as *RRS1* and *RPS6KA1*. (**D**) **Differential correlation of FOXO1 expression with cytosolic and mitochondrial ribosomal proteins.** *FOXO1* was differentially correlated to cytosolic (green) and mitochondrial (blue) RP genes. Each green or blue bar represents the correlation coefficient between *FOXO1* and one RP gene summarized from four sample groups of all tissue-disease combinations using the DerSimonian-Laird meta-analysis method. The red bars on both ends indicate the overall average, with p values computed by Student’s t-test. (**E**) ***FOXO1*** **differential expression at gene and sub-gene levels.** Differential *FOXO1* expression in both cell types is symbolized as two icons. Colors convey direction (green = down, red = up) and statistical significance of differential expression in RC disease. Triangles on the left are 5'-UTRs whose differential expression was more significant than the respective whole transcript average (see detailed keys in **Fig. 6**). See also **Fig. S6 in File S3**.

TF activities were evaluated by assessing the average transcriptional change of their target genes in RC disease **(File S1)**. When testing target genes that harbored TF binding sites within their promoters, a “muscle-up/FCL-down” pattern was most evident for *NRF2*, *ETS1*, and *ELK1* (**Fig. 5A**). *NRF2* responds to oxidative stress to regulate genes whose promoters contain anti-oxidant response elements [19], whereas both *ETS1* and *ELK1* belong to the Ets TF family that converge MAPK, ERK1, and PI3K signaling pathways [20]. Conversely, the most significantly “muscle-down/FCL-up” TFs were *TATA*, *AP4*, and *RREB1*, where *RREB1* is involved in Ras-mediated cell growth and differentiation [21]. The PPAR TF family also showed a consistent and significant “muscle-down/FCL-up” pattern, although FOXO family members had relatively insignificant overall change in RC disease. Interestingly, an interaction between PPAR binding sites and nearby TATA sites has been previously reported [22]. Indeed, we observed that genes containing both PPAR and TATA sites in their promoters were even more significantly downregulated than were genes containing only one of these two sites (**Fig. S3A in File S3**).

Testing target genes whose TF binding sites were located within their introns revealed a very different pattern (**Fig. 5B**). Here, the FOXO TF family was among the factors displaying the most significant “muscle-up/FCL-down” correlations in RC disease, whereas the PPAR TF family was not changed. It is unclear why TATA and the closely related TATA binding protein (TBP) both appeared in opposite positions when analyzed based on their binding sites falling into either the promoter (**Fig. 5A** **)** or intronic **(** **Fig. 5B**) region of genes, although a large fraction of TBP-binding sites has been reported to fall within introns [23]. Another TF whose binding sites correlated with a “muscle-up/FCL-down” pattern was *CREBP1*, which regulates transcription by modifying histone subunits [24]. Interestingly, two of the most significantly “muscle-down/FCL-up” TFs were *SP1* and *MAZR*, which are also involved in chromatin remodeling [25], [26]. Since histone modifications within introns have previously been linked to transcriptional regulation [27], [28], these results are suggestive that epigenetic dysregulation is involved in the cellular response to mitochondrial RC disease.

The TF screening procedure described above was cross-validated by analysis of a 15 base pair position weight matrix (PWM) of peroxisome proliferator response elements (PPREs) that are common to all PPAR family members [29]. This PWM is also highly similar to that derived from ChIP-seq data in human and mouse liver [30]. Searching the human reference genome for genes whose [−10 kb, 1 kb] promoters include matching sites with at least 95% similarity to this PWM identified 261 unique genes as potential PPAR targets (**File S1**). The differential expression in RC disease between these 261 target genes and all non-target genes was highly significant (p = 8.8×10^−10^ in muscle and p = 5.5×10^−35^ in FCLs) (**Fig. S3B in File S3**), thereby confirming that PPAR family TF activity is altered in RC disease.

### Cytoplasmic and Mitochondrial Ribosomal Protein Genes Show Concordant Dysregulation in an Inverse and Tissue-specific Fashion in Primary RC Disease

A striking pattern of differential expression of ribosomal protein (RP) genes in RC disease was evident in both muscle and FCLs (**Fig. 5C**). The 75 mitochondrial RP genes were generally upregulated in RC disease muscle (p = 5.5×10^−9^), as might be expected in cellular response to defective mitochondrial ATP generation. In contrast, the 60 cytosolic RP genes were generally downregulated in RC disease muscle (p = 2.0×10^−7^), as is consistent with impaired cell proliferation and growth that is typical of RC disease. An inverse pattern of differential expression for both RP gene groups was evident in RC disease FCLs. Since upregulating cytosolic RP genes is not likely to result in a sustained improvement in FCL growth in the setting of RC disease, we postulated this finding represented a byproduct of the same, albeit reversed, regulatory mechanism(s) that leads to downregulation of the cytosolic RP gene set in muscle. Such tissue-specific gene set co-regulation was suggestive that a central TF, or set of TFs, directly induced the RP gene set changes that we observed in primary RC disease.

The distinctive pattern of RP gene changes was not unique to our RC disease patient cohort. Upon our analysis of an independent public microarray data set comparing FCLs of 13 subjects with ATP synthase (complex V) RC deficiency to 9 healthy controls [31], we observed the same pattern of RP dysregulation among both cytoplasmic and mitochondrial RP genes (**Fig. S4A in File S3**). Similar to the changes we observed in human RC disease FCLs, significant mitochondrial RP gene set downregulation (p = 2.3×10^−15^) was also evident in public transcriptome data from mouse embryonic fibroblasts treated with the mTORC1 inhibitor, rapamycin (**Fig. S4B in File S3**). Furthermore, our analysis of public transcriptome data from a primary tissue (blood) of a homogenous population of mitochondrial disease patients affected with mitochondrial encephalopmyopathy lactic acidosis and stroke (MELAS) who harbor the m.3243A>G common mutation [32] revealed a similar RP dysregulation pattern as seen in RC disease FCLs (**Fig. S4C in File S3**).

Co-regulation of RP genes has been previously proposed [33]. In yeast, the nutrient-sensitive TOR-PKA pathway regulates RP gene transcription through *FHL1*, a forkhead-like TF that binds promoters of almost all RP genes. Although *FHL1* has no human homolog [34], co-regulation of RP genes might still exist in humans, perhaps in a more complex fashion, since shared regulatory motifs of RP genes are highly evolved across distant species [35]. The promoters of both cytosolic and mitochondrial RP genes are enriched for binding sites of *NRF2* and *YY1*, where *YY1* is an mTORC1-regulated TF that controls genes related to mitochondrial oxidative phosphorylation and insulin signaling [36] (**Fig. S5 in File S3**). However, there has not previously been a recognized role for these two TFs on the regulation of RP gene transcription.

Instead, our correlation analyses suggested that transcription of many RP genes correlated with transcription of *FOXO1*, a key TF mediating insulin sensitivity [37]. Calculating the combined correlation coefficient across four sample groups (two tissue types each studied in two disease statuses) showed that *FOXO1* expression in primary RC disease correlated positively with most cytosolic RP genes but negatively with most mitochondrial RP genes (**Fig. 5D**). In addition, we discovered that *FOXO1* gene expression was downregulated in RC disease muscle and upregulated in RC disease FCLs, especially at its 5′-UTR (**Fig. 5E**).

The observed expression correlation between *FOXO1* and RP genes could result from several different mechanisms, including direct binding and regulation, indirect regulation, or co-regulation by a common upstream regulator (such as in the AKT signaling pathway). In Drosophila, the *FOXO* homolog (dFOXO) represses the expression of genes related to both mitochondrial [38] and cytosolic [39] ribosomes, and further reduces ribosomal biogenesis via transcriptional downregulation of *myc* [40]. Although direct regulation of RP genes by *FOXO1* cannot be proven with the current data set, supporting evidence is provided by the finding that 11 mitochondrial RP genes whose introns contain human/mouse/rat conserved *FOXO1* binding sites were unanimously upregulated in RC disease muscle (**Fig. S6A in File S3**). Cytosolic RP genes are less likely to be direct *FOXO1* targets, since their overall correlation with *FOXO1* was not as strong as seen for mitochondrial RP genes (**Fig. 5D**) and only one of them had an intronic *FOXO1* binding site. However, other forkhead TFs did show strong expression correlation with cytosolic RP genes. *FOXK1*, a regulator of muscle development [41], and *FOXC2*, a mediator of PKA signaling and insulin sensitivity [42], [43], were more significantly correlated with cytosolic RP genes than was *FOXO1*, although neither of these TFs were strongly associated with mitochondrial RP genes (**Fig. S6B in File S3**). Thus, we postulate that one or more TFs, including *FOXO1*, collaboratively regulate the concordant differential expression of cytosolic and mitochondrial RP gene sets in primary RC disease.

### RC Disease Alters Transcript Processing

Given the differentially expressed sub-gene regions detected for *FOXO1*, we investigated the possibility that primary RC disease more globally affects the differential expression of sub-gene regions by alternative RNA processing. Individual microarray probes were assigned to non-overlapping UTR or exon probesets that were summarized for probeset-level group difference analysis by SAM methods (**File S1**). If a gene had multiple probesets for the same UTR or exon, the probeset with the smallest p value was selected and adjusted for multiple testing by Bonferroni correction. A probeset indicated an alternative event if its adjusted p value was less than 0.05, and the p value was either significantly smaller than the gene-level p value or the probeset was changed in the opposite direction from the gene-level change. A large number of genes satisfied these criteria (**Table 1**). In particular, 6,440 genes in muscle and 5,982 genes in FCLs were found to have at least one alternative expression event in RC disease. DAVID analysis of the 2,626 genes common to both muscle and FCLs demonstrated highly significant enrichment of genes known to undergo alternative splicing (p = 4.7×10^−16^).

**Table 1:** Transcriptional changes of sub-gene regions in RC disease.

Gene Sub-Region	5′-UTR		3′-UTR		Exon		Antisense	
Cell Type	Muscle	FCL	Muscle	FCL	Muscle	FCL	Muscle	FCL
**Total** **Gene # analyzed**	15,406		16,873		20,518		4,141	6,200
**# Genes Upregulated**	180	1,672	984	525	1,453	1,401	197	1,040
**# Genes Downregulated**	2,012	784	732	1,125	2,029	1,937	286	540

The total number of genes analyzed in each gene sub-region is indicated, with delineation of the number of genes in which the indicated sub-gene region (5′ UTR, 3′ UTR, or Exon) or antisense transcription was significantly upregulated or downregulated in each cell type in primary RC disease relative to controls. Only effective probesets were included in analysis of antisense transcripts to reduce noise, since the majority of antisense transcripts were not present in both cell types. UTR, untranslated region. FCL, fibroblast cell line.

**Figure 6:**
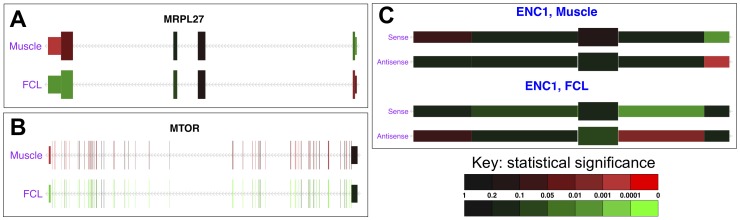
Distinctive examples of genes showing significant sub-gene changes in RC disease. Each vertical bar represents a microarray probeset, while narrower bars on gene ends indicate UTRs. Arrowheads indicate the direction of transcription. (**A**) ***MRPL27***, a mitochondrial RP gene, had significant changes at both its 5' and 3'-UTRs toward opposite directions in RC disease both cell types. Consequently, it was not identified as a DEG by whole transcript level analysis. (**B**) ***mTOR*** was consistently and significantly changed across the entire gene in RC disease. (**C**) ***ENC1*** had modified antisense transcription in RC disease. The opposite direction of expression changes within its 5'-UTR suggested that the ENC1 antisense transcript might be functional. See also **Fig. S7 in File S3**.

Distinctive patterns of sub-gene level differential expression were evident for many genes having clear biological relevance to RC disease. For example, the mitochondrial RP gene, *MRPL27*, was not significantly changed according to the gene-level analysis but showed opposite and significant 5′ and 3′-UTR changes in RC disease muscle and FCLs (**Fig. 6A**). Conversely, *mTOR* showed consistent change across the whole gene (**Fig. 6B**). Considered together with many other examples of probeset-level differential expression patterns that we observed in individual genes (**Fig. S7 in File S3**), these findings collectively indicate that a highly complex regulatory disturbance of RNA processing occurs in primary RC disease.

#### Antisense transcription is altered in primary RC disease

RNA processing is also known to involve antisense transcription [44]. The Affymetrix microarray platform used in this study included 26,530 probesets assigned to the antisense strand of known transcripts. As expected, antisense transcripts had lower abundance than sense transcripts on average, although 5′-UTRs and exons of non-coding genes had higher antisense transcription than coding exons and 3′-UTRs (**Fig. S8 in File S3**). Since antisense transcription is not considered to be a generic event, we further analyzed 5,508 muscle and 9,083 FCL effective antisense probesets that had significantly higher microarray signals above background (**File S1**). A large number of genes having modified antisense transcription in RC disease were identified in both tissues, especially in FCLs (**Table 1**).

Baseline abundance and RC disease-control differences were both positively correlated between sense and antisense transcripts (**Fig. S9 in File S3**), supporting the prior suggestion that most antisense transcripts represent transcriptional noise [45]. However, some antisense transcripts had changes in RC disease that were opposite to their sense counterparts, and might have a potential role in repressing gene expression [46]. For example, *ENC1*, a regulator of *NRF2* during oxidative stress response, had opposite changes of sense and antisense transcription at its 5′-UTR in both tissue types (**Fig. 6C**), which is indicative that antisense *ENC1* transcripts might be functional. Considered together with additional examples of genes having differential antisense transcription (**Fig. S10 in File S3**), these data are suggestive that antisense transcription is another regulatory system that is affected by primary RC disease.

#### RC disease alters stability of 3′-UTRs with AU-rich elements (AREs)

The direction of UTR expression changes in RC disease was highly unbalanced (**Table 1**), which is unlikely to have resulted from bias introduced by microarray experiments or data processing for two reasons. First, all types of probesets including exons and UTRs were normalized together. Second, the tissue-specific pattern of UTR dysregulation was consistent with the recurrent theme of tissue-specific effects of RC disease. Therefore, we conclude that RC disease muscle shortens or destabilizes 5′-UTRs and lengthens or stabilizes 3′-UTRs, whereas the opposite trend occurs in FCLs. Since UTRs are known to influence mRNA transport, localization, stability and translation efficiency [47]–[49], this observation strongly supports our earlier finding that RNA processing genes were highly enriched in “muscle-up/FCL-down” DEGs in primary RC disease (**Fig. 4B**).

**Figure 7:**
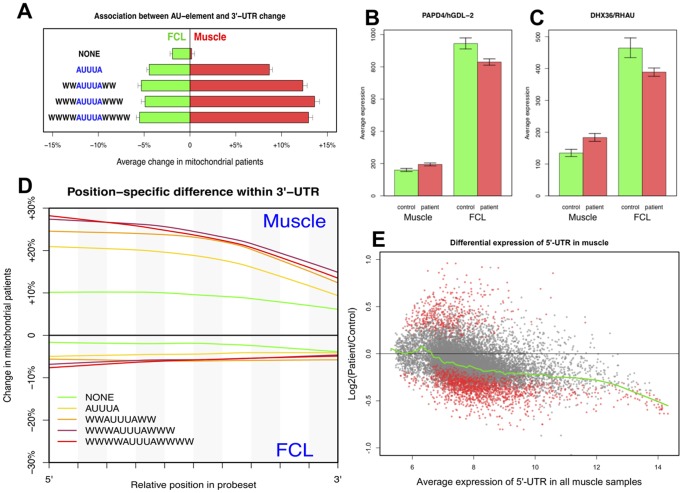
Differential UTR expression in RC disease. (**A**) **Association between AU-rich elements and 3′-UTR changes in RC disease.** All genes were split into five exclusive groups based on the presence and type of AU-rich elements (ARE) in their 3′-UTRs. Genes in muscle without ARE had little change of their 3′-UTR, while those with long ARE motifs showed the most significant average increase. The reverse trend of lesser magnitude occurred in RC disease FCLs. Only effective probesets having expression significantly higher than background were analyzed. (**B**) ***PAPD4*** encodes the GDL-2 protein that acts as a cytoplasmic poly(A) polymerase, and was dysregulated by RC disease in a fashion consistent with its overall 3′-UTR changes in both cell types. (**C**) ***DHX36***, also known as *RHAU*, enhances RNA decay by binding to AU-rich elements (AREs) in 3′-UTRs. Interestingly, DHX36 dysregulation in RC disease positively correlated to changes in 3′-UTRs containing AREs, which likely reflects its novel role in the regulation of RNA structure and synthesis. (**D**) **Position-specific differential expression of 3′-UTRs in RC disease.** Probes mapped to 3′-UTRs were assigned to 1% intervals from the 5′ to 3′ ends. Since 3′-UTR degradation starts from the 3′-end, the increasing difference from the 3′ to 5′ indicates a gradually decreased pace of 3′-UTR degradation occurs in RC disease. (**E**) **Relative 5**′**-UTR change in RC disease is dependent on the baseline absolute abundance of 5**′**-UTR in muscle.** Significantly changed 5′-UTRs (p<0.05) are highlighted in red, with the green line generated by Lowess smoothing. This plot demonstrates the nearly unanimous downregulation in RC disease of 5′-UTRs with the highest baseline transcription levels. See also **Fig. S12 in File S3**.

3′-UTR stability is regulated by binding of regulatory proteins to AU-rich elements (AREs) located in the 3′-UTRs [50]. All AREs include a core motif, AUUUA, but often exhibit a more specific form such as WWWAUUUAWWW, where W is an A or U. Our analyses identified a strong association between the presence of AREs and the 3′-UTR changes induced by RC disease, particularly in muscle. ARE-containing gene lists were downloaded from the AREsite database [51] and classified into 5 mutually-exclusive groups based on the complexity of ARE motifs, from the basic AUUUA pentamer to the most complex 13mer (**File S1**). The average 3′-UTR changes of these gene groups in RC disease muscle (**Fig. 7A**) revealed: (1) genes without a 3′-UTR ARE had almost no change; (2) genes having the basic AUUUA motif were increased by an average of approximately 8% (p = 7.7×10^−73^); and (3) genes having extended ARE motifs were more significantly increased by an average of more than 16% (p = 2.6×10^−174^). The inverse pattern, albeit less pronounced, was evident in RC disease FCLs (p = 1.1×10^−11^ and 2.7×10^−22^, respectively). Collectively, these results suggest that the 3′-UTR changes observed in RC disease are predominantly caused by a disturbance of ARE-based 3′-UTR regulation.

Genes involved in 3′-end mRNA processing were indeed enriched in “muscle-up/FCL-down” DEGs (p = 0.003), such as *PAPD4* that encodes GLD2, a cytoplasmic poly(A) polymerase [52]. GLD2 polyadenylates the 3′-end of mRNAs and specifically elongates AU-rich 3′-UTRs to improve their stability [53]. *PAPD4* had higher baseline transcription in FCLs than in muscle, but was significantly changed by RC disease in both tissues (**Fig. 7B**). While most genes known to directly bind to AREs showed insignificant or marginally significant changes, a notable exception was *RHAU/DHX36*, which facilitates mRNA deadenylation and decay [54]. Surprisingly, the direction of *RHAU* change was “muscle-up/FCL-down” (**Fig. 7C**), which was unexpected given the higher 3′-UTR level in RC disease muscle. This conflicting result may relate to the recently recognized roles of *RHAU* in regulating RNA quadruplex structures [55] and nuclear RNA synthesis [56].

UTR changes in RC disease might result from alternative transcriptional initiation, termination, and splicing, and/or from a modified rate of mRNA degradation. If the latter were the predominant factor, UTR ends would display larger group differences than their central regions. Position-dependent differential expression within UTRs was evaluated by categorizing individual probes based on their relative locations within UTR probesets (**File S1**). Results showed that 3′-UTRs did have a significantly reduced degradation ratio in RC disease muscle, whereas the trend was reversed and less pronounced in FCLs (**Fig. 7D**). Thus, a modified rate of 3′-UTR degradation likely contributes to the 3′-UTR changes in RC disease.

#### 5′-UTR changes in RC disease correlate with baseline transcript abundance

Unlike the 3′-UTRs, 5′-UTRs displayed little position-dependent expression differences in RC disease in either tissue (**Fig. S11 in File S3**). We therefore investigated whether the observed differences in 5′UTR length was influenced by the 5′ terminal oligopyrimidine (TOP) motif that is located immediately after transcriptional start sites and is a known regulator of protein translation that is found in most RP genes [57]. Interestingly, mTORC1 preferentially regulates translation of TOP-containing mRNAs [58]. However, no association was observed between the presence of a TOP motif and 5′-UTR changes in RC disease, likely because TOP motifs do not regulate translation by modifying 5′-UTR transcription or degradation. Interestingly, the baseline abundance of 5′-UTRs relative to their whole gene transcript levels was generally higher in muscle than in FCLs (**Fig. S12 in File S3**), which may reflect the greater complexity of *in vivo* post-transcriptional regulations. A strong association was detected between the baseline abundance of 5′-UTRs in muscle and their downregulation in RC disease (**Fig. 7E**). Since translation initiation factors are more efficient at binding and scanning shorter 5′-UTRs [59], we postulate this might represent a reactive mechanism by which cells attempt to compensate for poor cellular growth caused by primary RC disease.

#### Nutrient-sensing signaling network changes in primary RC disease

**Figure 8:**
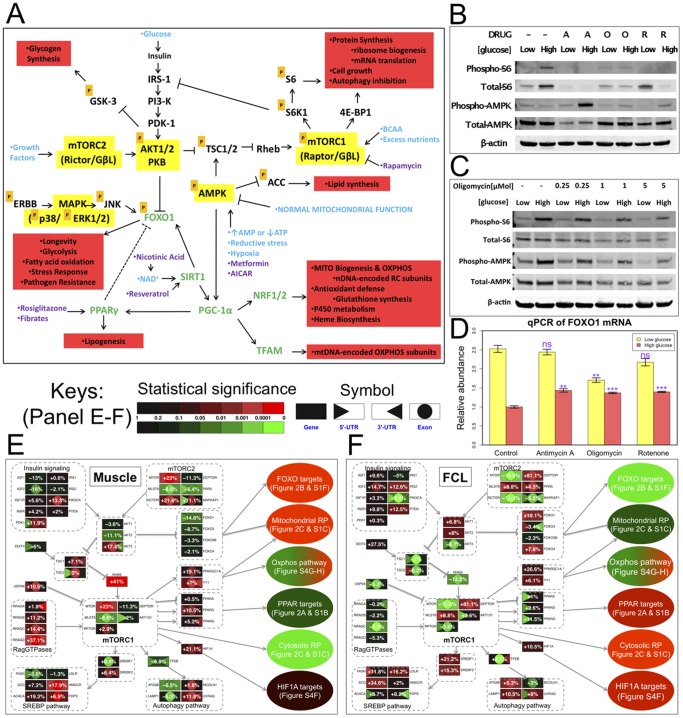
Primary RC dysfunction transcriptionally and post-transcriptionally dysregulates the integrated nutrient-sensing signaling network. (**A**) **Integrated overview modeling general interactions between central nutrient-sensing signaling pathways.** Arrows and bars convey activating and inhibiting effects, respectively. TFs, physiologic signals, and drugs known to modulate this pathway are indicated in green, blue, and purple font, respectively. “P” indicates pathway components whose activity is modulated by phosphorylation. Red boxes detail physiologic effects. (**B**) **Oligomycin-based pharmacologic RC inhibition in human FCLs alters mTORC1 and AMPK pathway activities.** To confirm primary mitochondrial RC dysfunction was sufficient to alter mTORC1 signaling, FCLs from a healthy individual were treated in DMEM medium containing 20% fetal bovine serum for 24 hours and either low (1 g/L or 5 mMol) or high (4.5 g/L or 25 mMol) glucose, with either the complex V inhibitor, Oligomycin, (“O”, 5 uMol), an AMPK activator, AICAR (“A”, 2 mMol), or the mTORC1 inhibitor, rapamycin (“R”, 100 nMol). Regardless of glucose concentration, expression of a standard readout of mTORC1 pathway activity, phospho-S6 protein level, was reduced by oligomycin treatment, while increased phospho-AMPK expression was evident in high glucose media. (**C**) **Oligomycin-based alteration of mTORC1 and AMPK pathway activities is dose- and nutrient-dependent.** The same control FCLs as in (B) were treated for 24 hours in either low (1 g/L or 5 mMol) or high (4.5 g/L or 25 mMol) glucose media with oligomycin in doses ranging from 0.25 uMol to 5 uMol. A general trend is evident of overall reduced phospho-S6 and phospho-AMPK at increasing doses of oligomycin. See also **Fig. S13 in File S3**. (**D**) **Pharmacologic RC inhibition increases** ***FOXO1*** **expression in high-glucose conditions.** FCLs were treated for 24 hours in either low (1 g/L or 5 mMol) or high (4.5 g/L or 25 mMol) glucose media with a RC inhibitor targeting either complex I (rotenone, 0.1 uMol), III (antimycin A, 0.5 uMol), or V (oligomycin, 1 uMol). As expected, *FOXO1* expression was inhibited by high-glucose media. All three RC inhibitors increased *FOXO1* relative expression in high-glucose conditions, whereas *FOXO1* expression was decreased by oligomycin in low-glucose media. (**E–F**) **Integrated view of detailed gene and sub-gene level transcriptional alterations in RC disease among central nutrient-sensing signaling network modulators.** Their major downstream targets and pathways were presented in separated plots. An overall inverse expression pattern is evident in RC disease between (**E**) skeletal muscle and (**F**) FCL.

A central role of an integrated nutrient-sensing signaling network in mediating transcriptional, post-translational, and translational dysregulation in primary RC disease seemed likely based on the specific genes we had found to be implicated through the diverse systems biology level analyses already described. Indeed, cellular inputs related to the bioavailability of major nutrients (i.e., glucose, amino acids, fatty acids) as well as general energy status are widely recognized to converge on central signaling mediators such as mTORC1, FOXO, AMPK, and PPAR (**Fig. 8A**). These mediators regulate the main cellular effector pathways, which include key intermediary metabolic pathways, to influence the balance of cell growth and death.

Primary RC dysfunction has long been postulated to reduce ATP, increase AMP, and stimulate AMPK [60]. However, a central role of mTORC1 in effecting the cellular response to primary RC dysfunction, as was evident both in our data from a heterogeneous population of primary RC disease subjects and upon our analyses of public data from a homogenous population of individuals with RC complex V disease (**Fig. S4C in File S3**), has not been widely recognized. To confirm that mTORC1 activity is indeed altered by primary RC dysfunction, we treated healthy human FCLs with high (5 uMol) concentration of the RC complex V inhibitor, oligomycin, which decreased expression of phospho-S6 protein, a major indicator of mTORC1 activity, in a nutrient (glucose)-dependent fashion (**Fig. 8B**). Additional analyses of a range of oligomycin doses (0.25 uMol to 5 uMol) both in healthy human FCLs (**Fig. 8C**), as well as analysis of three different RC complex inhibitors in healthy human podocytes (**Fig. S13 in File S3**), confirmed that direct RC inhibition alters mTORC1 and AMPK activities in a time, dose, and nutrient-dependent fashion. High dose oligomycin treatment in human fibroblasts also upregulated *FOXO1* transcript levels in high-glucose conditions (**Fig. 8D**), which is similar to the increase in *FOXO1* expression seen in primary (genetic-based) RC disease FCLs (**Fig. 5E**). These results are suggestive that the severity and duration of RC inhibition, together with the general cellular nutrient availability, can differentially influence the activity of the integrated nutrient-sensing signaling network that controls cellular proliferation and death.

The inherent complexity of the regulatory network mediating the cellular response to primary RC dysfunction was evident upon detailed dissection of expression and activity levels both among central members of the integrated nutrient-sensing signaling network and their downstream targets (**Fig. 8E–F**). The same tissue-specific pattern of opposing transcriptional changes described earlier at the global level was again highly prominent in RC disease muscle (**Fig. 8E**) and FCLs (**Fig. 8F**). Furthermore, individual network members demonstrated a wide range of specific responses to RC disease that involved different regulatory systems. For example, whereas transcription of genes like *RHEB* and *MTOR* was consistently modified across the whole gene, *FOXO1* was most significantly modified at its 5′-UTR. Post-translational regulation was also apparent. For example, S6 expression was changed at the level of protein modification (**Fig. 8B–C**), while altered activity of PPAR family TF activity was suggested by concordant changes in expression of their target genes (**Fig. 8E–F**).

Further evidence for dysregulated translation in RC disease was reported by Danielson et al., who studied the transcriptome of Leber’s hereditary optic neuropathy (LHON), a common mitochondrial RC disease caused by point mutations of mtDNA-encoded RC complex I subunits [61]. Pairwise comparisons were performed both in cybrid osteosarcoma cell lines that were constructed to harbor mtDNA both with and without LHON mutations, and in lymphoblastoid cells from control subjects and LHON probands. Nine differentially expressed genes were identified by both comparisons, including increased aldose reductase, and decreased *Raf1* and Sialyltransferase 1 (*Siat1*). Based on this result, a model was proposed within which increased ROS and aldose reductase expression decrease expression of pro-survival and proliferation genes, such as *Raf1* and *Siat1*, thereby leading to apoptosis as is observed in LHON. While aldose reductase was slightly up-regulated on average in both our RC disease muscle and FCL datasets, this change was not statistically significant in either tissue as only some of the RC samples had higher aldose reductase expression. However, the Danielson study also identified 4 genes that encode eukaryotic translation initiation factors that were differentially expressed in LHON subject lymphoblastoid cells, as well as upregulation of 3 genes that encode translation elongation factors. Analysis of our data set demonstrated that all 3 translation elongation factor genes were downregulated in RC disease muscle and upregulated in RC disease FCL. Similar findings in three different RC disease tissues (muscle, FCLs, and lymphoblastoid cell lines) data provide further support for dysregulated translation in RC disease, where the direction of transcriptional response once again is highly tissue specific.

**Figure 9:**
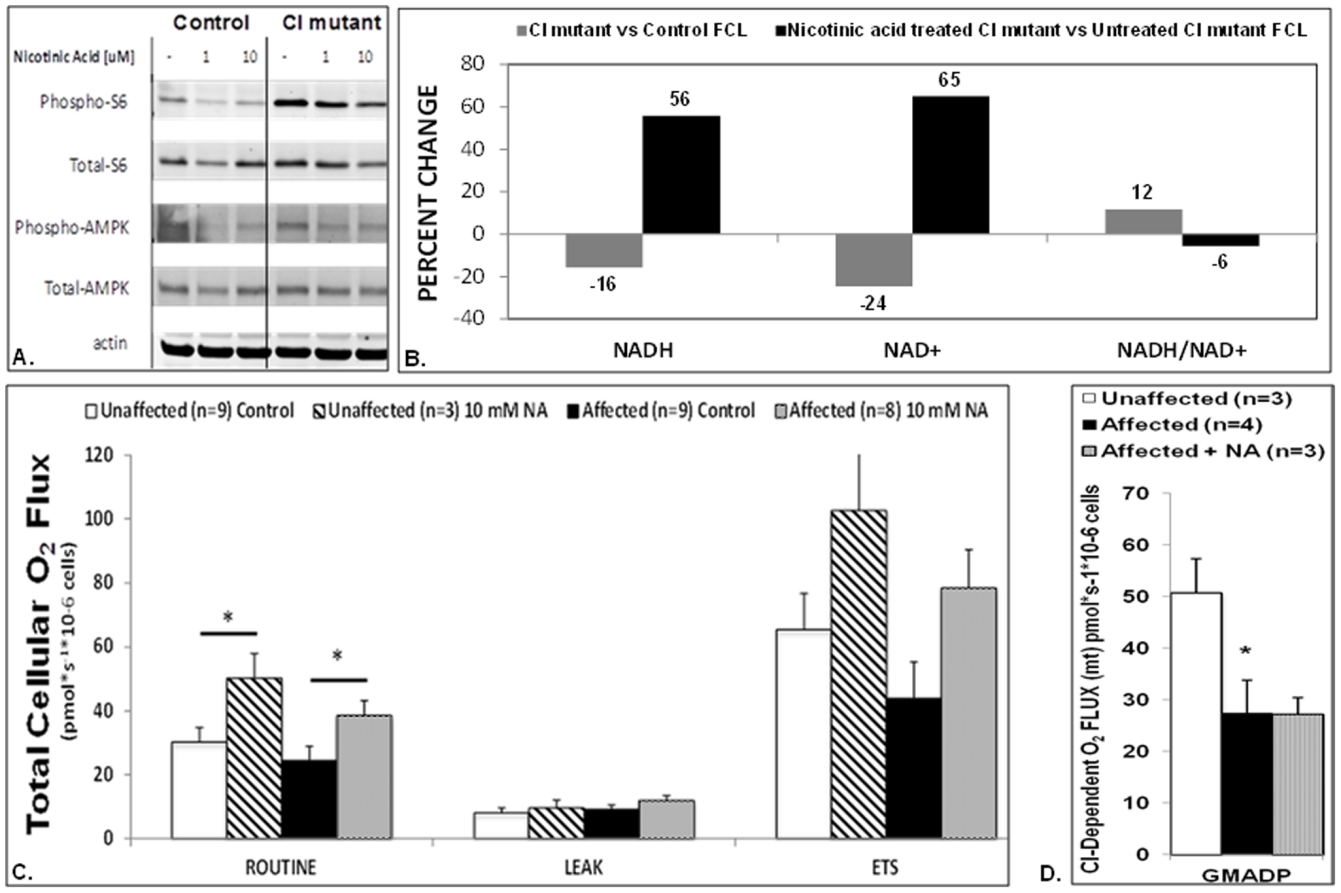
Nicotinic acid treatment in a complex I mutant FCL reverses mTORC1 and AMPK activation and rescues cellular redox poise and respiratory capacity. (**A**) Upregulated P-S6 and P-AMPK expression in the Q1039 CI mutant FCL was mitigated in a dose-dependent fashion with 24 hour nicotinic acid treatment (lanes 5–6). Cells were grown in the same low glucose concentration (5 mMol) as was used for microarray studies. The proband has Leigh syndrome caused by known pathogenic mtDNA mutations in two complex I subunits, ND4 and ND6 (**Fig. 1**). (**B**) Total cellular deficiency in both NADH and NAD^+^ (as measured by HPLC as pMol/mg protein), along with an increased NADH/NAD^+^ ratio, was evident in the same CI mutant FCL relative to control FCL (gray). NADH and NAD^+^ cellular levels, as well as NADH/NAD^+^ redox balance, were all reversed by 24 hour nicotinic acid (10 mMol) treatment in CI mutant FCLs relative to untreated CI mutant FCLs (black). Percent change in mean [NAD^+^], [NADH], and calculated NADH/NAD^+^ ratios are shown, as determined in 3 biologic replicates per condition. (**C**) Intact FCLs from the same Q1039 CI-mutant proband (black) showed no significant change in total cellular routine (basal) respiratory capacity relative to unaffected control (white) oxygen flux as measured by high-resolution respirometry (Oxygraph-2K, Oroboros). However, nicotinic acid (10 mmol) treatment for 24 hours significantly increased total cellular respiratory capacity both in control (slash) and affected (dotted) cells. *, p<0.05 by ANOVA followed by paired t-test. Maximal (electron transport system, ETS) respiration was significantly greater than routine respiration for three of the conditions studied (CI mutant cells with no treatment, p = 0.0033; CI mutant cells with nicotinic acid treatment, p = 0.007, and control cells with no treatment, p = 0.002 by paired t-tests. No significance was reached when comparing basal and maximal respiratory for the nicotinic acid treated control cells, possibly due to small sample size (n = 3, p = 0.1126). Leak (background or non-mitochondrial) respiration showed no difference in any condition. Bars indicate mean±SEM. (**D**) Digitonin-permeabilized fibroblast analyses (right panel) reliably discriminated the specific complex site of RC dysfunction in affected CI mutant (Q1039) cells (black), as complex I-dependent (“GMADP, glutamate+malate+ADP) cellular respiratory capacity was significantly reduced in CI mutant FCLs relative to unaffected control FCLs (white). The genetic-based deficiency in CI-dependent respiratory capacity was not improved by 24 hour treatment with nicotinic acid (10 mMol, gray). Bars indicate mean±SEM. *, p<0.05.

To assess the possible therapeutic implications of our observation that central nodes of the nutrient sensing signaling network were modified in primary RC disease, we investigated the mTORC1 and AMPK activities of a FCL from a mitochondrial disease subject (Q1039) who has Leigh syndrome caused by pathogenic mtDNA mutations in two complex I subunit genes (*ND4* and *ND6*). Her FCLs grown in low glucose (5 mMol) had upregulated P-S6 and P-AMPK expression (**Fig. 9A**), as was consistent with our finding of increased cytosolic ribosome expression among human RC disease FCLs grown under the same conditions (**Fig. 5C**). Remarkably, treating her FCLs with nicotinic acid, which activates PPARs at least in part via receptor stimulation [62]–[64] and serves as a precursor for biosynthesis of NAD^+^ that can enhance sirtuin activity [65], [66], also reduced her upregulated mTORC1 and AMPK activities in a dose-dependent fashion (**Fig. 9A**). Furthermore, her FCLs manifest a significant reduction in both total cellular NADH and total cellular NAD^+^ content along with increased NADH:NAD^+^ redox balance, which were all rescued by 24 hour treatment with 10 mMol nicotinic acid (**Fig. 9B**). Most importantly, 10 mMol nicotinic acid treatment for 24 hours improved total cellular respiratory capacity in intact cells (**Fig. 9C**), a phenomenon which high-resolution respirometry studies in digitonin-permeabilized cells revealed were not due to correction of her inherent defect in complex I-dependent respiration (**Fig. 9D**). Overall, these *in vitro* studies confirm that multiple nutrient-sensing signaling nodes may be dysregulated in primary RC disease, and suggest that therapies targeted to central nodes may reverse changes in the integrated signaling network, with resultant improvement in overall cellular function despite persistence of the underlying genetic-based primary RC defect.

## Discussion

Primary RC disease causes a dysregulated transcriptome pattern that is evident in both skeletal muscle and FCLs across a diverse spectrum of specific RC complex defects, genetic etiologies, and clinical disease manifestations. Overall, mitochondrial RC disease muscle displays a common transcriptional and post-transcriptional response to its primary RC dysfunction that constitutes a pathogenic molecular signature that is characterized by a reduction of cytosolic ribosomes, increase in mitochondrial ribosomes, decrease in 5′-UTR transcription to improve translational efficiency, and prolongation of 3′-UTR length to stabilize mRNAs. This pattern reflects a common downstream response to inefficient RC functions, which provides a transcriptional profile of RC disease that may be of potential utility as a diagnostic aid in the clinical setting. Identification of novel adaptive mechanisms at transcriptional and post-transcriptional levels to primary RC disease can also serve as a means to monitor primary RC disease progression or even response to therapy in clinical trials. Thus, these findings support the utility of applying a systems biology approach to the investigation of mitochondrial disease [67].

The striking but globally opposite changes seen in RC disease muscle and FCLs is consistent with the very different energy requirements of these two tissue types. We postulate that increased expression of mitochondrial ribosomal proteins occurs in muscle as attempted cellular compensation to their primary inability to generate sufficient ATP necessary to support basic muscle function. In contrast, RC disease FCLs appear to minimize mitochondrial-dependent functions, perhaps as a means to conserve energy. Similarly, a tissue-specific response to mTOR knockout is known to occur as a means to maintain whole-body energy homeostasis [68]–[70]. As the primary defect in all of the patients studied originates in the respiratory chain, all transcriptome changes are by definition secondary adaptations that do not all necessarily achieve similar levels of functional efficacy in different tissues. However, deriving the primary skeletal muscle samples utilized for transcriptome analysis from the same muscle biopsy specimens in which the patient’s primary RC deficiency was biochemically established lends support to the biologic conclusions drawn from transcriptome profiling in RC disease muscle.

The opposite direction of transcriptome changes identified in the patients’ FCLs can arguably be impacted by several different variables surrounding tissue culture conditions, as we specifically demonstrate with regard to nutrient status. However, analyzing cells under different nutrient conditions may itself prove of clinical utility in understanding whether a key phenotype of mutant cells (such as respiratory capacity or profiling activity of central nodes in the nutrient-sensing signaling network) may be rescued or exacerbated by nutrient conditions in a pattern suggestive of primary RC dysfunction. Such analyses could be indicative of potential nutrient-based *in vivo* therapeutic modulation for nutrient-sensing signaling network alterations (eg, providing a given patient a high-glucose diet to mitigate the effects of their primary RC disease).

Our data set reduces potential effects of variability associated with individual disease progression, since ten of the muscle biopsy and FCL samples were derived from the same individuals. Interestingly, our analysis of public transcriptome data from a primary tissue (blood) of a homogenous population of MELAS patients revealed a similar response in terms of ribosomal protein expression as we observed in RC disease FCLs [32]. In addition, a transcriptome study in primary dopaminergic neurons from Parkinson’s disease patients brains identified AKT, FOXO, and mTOR signaling as playing a central role in modulating the transcriptional effects of apparent upstream mitochondrial RC dysfunction [71], with reported direction of alterations in that study showing direct concordance to our findings in human RC fibroblast cell lines. These similar findings in primary tissues of RC disease patients are suggestive that the opposite patterns of transcriptome changes observed in our muscle and FCL data sets may relate to biologic differences between different tissues types and not solely to artifacts of tissue culture. Moreover, recent studies have provided validation for the idea that FCLs provide relevant information about *in vivo* mitochondrial biology. AICAR, which activates AMPK, was recently identified as a compound with therapeutic potential in a patient FCL model of RC complex I disease [72] and subsequently shown to partially restore motor function in a mouse model of mitochondrial RC complex IV disease [73]. Our own data suggests that nicotinic acid treatment in a complex I patient FCL can reverse dysregulation of multiple nutrient-sensing signaling network nodes, and may have therapeutic value in improving cellular redox poise and respiratory capacity. Thus, despite inherent limitations of cell culture, FCLs from mitochondrial RC disease patients are considerably less invasive to obtain compared to muscle tissue and offer an important tool in which to better understand causes and therapies for mitochondrial disease.

While all factors underlying the observed tissue-specific response to primary RC dysfunction are thus not currently understood, recognition of their existence implies there are likely a small number of central signals or molecular switches that modulate such inverse tissue responses and underlie much of the biological complexity that occurs in primary mitochondrial RC disease. Indeed, our prior work investigating therapies in the *Pdss2* mouse model of primary mitochondrial Coenzyme Q deficiency [5] showed that a single pharmacologic agent that does not rescue mitochondrial respiratory capacity can nonetheless reverse both the clinical disease phenotype and the central signaling alterations in PPAR pathway genes that clearly are changed in different directions in different tissues of the same animal. Since correcting the initiating gene mutations in mitochondrial RC diseases is currently not possible, identifying the proximal signaling mechanisms that sense and respond to mitochondrial RC dysfunction may allow reversal of many downstream cellular alterations that contribute to clinical features of mitochondrial diseases.

An integrated nutrient-sensing signaling network is clearly involved in effecting the cellular response to primary RC disease. Indeed, the most significantly upregulated single gene in RC disease muscle was *RHEB*, which is a direct regulator of mTORC1 function. The transcriptional upregulation of cytosolic RPs seen in the FCL dataset from primary RC disease subjects, both in our study and in multiple public data sets, was supported by significantly upregulated phospho-S6 protein expression in RC complex I mutant FCLs, which suggest that mTORC1 activity is substantially altered in primary RC disease cells, and clearly influenced by nutrient supply. Pharmacologic inhibition of the respiratory chain in human fibroblasts and podocytes also altered mTORC1 activity and AMPK activity, in a fashion dependent on the duration and degree of RC inhibition as well as on general nutrient status. Collectively, these data suggest that an mTORC1-centered regulatory signaling network that also involves AMPK, FOXO, sirtuin, and PPAR family members plays an essential role in mediating the cellular response to primary mitochondrial RC disease. Thus, recognition of a central role of the integrated nutrient-sensing signaling network in the cellular response to primary mitochondrial RC disease may offer novel therapeutic targets for this currently incurable albeit heterogeneous class of genetic disease.

Improvement in overall cellular function despite persistence of the genetic-based primary RC defect in the RC complex I mutant fibroblast line treated with nicotinic acid is particularly intriguing, especially considering that this treatment also attenuated the upregulated mTORC1 activity evident in this subject’s cells. Our rationale for testing nicotinic acid was two-fold. First, we had previously observed that PPAR and its targets were downregulated in a primary RC deficient mouse model and that disease amelioration in that model occurred with a drug (probucol) that we found to specifically increase both PPAR transcription factor and PPAR target gene expression [5]. Therefore, we studied nicotinic acid to test whether targeted PPAR stimulation, as is a known effect of nicotinic acid [62]–[64], would also be therapeutic in this human cell model of primary RC deficiency. Second, nicotinic acid also serves as a precursor for NAD^+^ [65], a cosubstrate for sirtuin enzymes, including SIRT3, which regulates multiple mitochondrial enzymes via deacetylation [74], and SIRT1, which promotes mitochondrial biogenesis via deacetylation and activation of PPARγ coactivator 1α (PGC1α) [66], [75]. Since our human tissue transcriptome data suggested that these same signaling pathways, along with mTORC1 and other related factors, move in a coordinated fashion during mitochondrial dysfunction, we hypothesized that correcting aspects of this dysfunctional signaling network with nicotinic acid would also reduce mTORC1 signaling toward normal. Our findings are consistent with prior reports that PPAR activation (using rosiglitazone) or SIRT1 activation can both suppress mTORC1 signaling [76], [77]. Thus, while the precise mechanism is not yet evident, it is clear that there are interconnections between central nutrient-sensing signaling pathways that play important roles in both responding to and effecting RC dysfunction. Further, since nicotinic acid is an NAD^+^ precursor and PPAR agonist, rather than a presumed electron donor, we would not expect it to correct complex I enzyme activity in a known complex I deficient cell line that harbors two clearly pathogenic CI subunit mutations. Indeed, the data presented confirms that complex I-dependent respiratory capacity as assessed using malate plus glutamate as substrates in permeabilized cells is not rescued with nicotinic acid. Similarly, nicotinic acid does not reduce the upregulated complex II-dependent respiratory capacity that occurs in the complex I deficient permeabilized cell line when using succinate as a substrate (data not shown). Nonetheless, nicotinic acid treatment does improve total cellular respiratory capacity. This may indicate that the cells have adapted to culture such that complex I activity is no longer strictly limiting under basal conditions. In particular, cultured cells are able to derive a large proportion of their ATP from glycolysis, rather than oxidative phosphorylation. An existence of excess respiratory capacity, even before nicotinic acid treatment, is evidenced by an increase in oxygen consumption rate when the complex I mutant subject’s fibroblasts were acutely uncoupled by FCCP. Interestingly, this suggests that mitochondrial respiration might be limited by signaling defects, rather than the inherent respiratory capacity of complex I, in the complex I mutant subject’s fibroblasts. It is also possible that nicotinic acid treatment increases respiration by inducing a shift toward fatty acid oxidation. Fatty acid oxidation is less reliant on complex I activity than is glucose oxidation due to the flow of reducing equivalents through the electron transfer flavoprotein [78]. Because of this, generating the same amount of ATP and consuming a similar amount of oxygen requires less complex I activity when fat is the fuel source. There are two hypothesized mechanisms by which the switch to fat oxidation might occur: [1] Increased NAD^+^ availability could increase the activity of sirtuin enzymes, which deacetylate multiple substrates to favor fatty acid oxidation, or [2] PPAR activation by nicotinic acid might directly increase the expression of genes involved in fat metabolism. As discussed above, each of these mechanisms are also known to potentially suppress mTORC1 signaling.

Overall, this work identifies a common signaling network response to mitochondrial RC disease that extends across different tissue types and can be identified despite heterogeneous molecular and biochemical etiologies of individual mitochondrial RC disorders. The molecular effects of mitochondrial RC disease in skeletal muscle involve global genome transcriptional alterations that implicate both transcriptional and post-transcriptional deregulation. The specific major components of this molecular signature include decreased transcription of cytosolic ribosomal proteins that is suggestive of reduced anabolic processes, increased transcription of mitochondrial ribosomal proteins, globally shorter 5′-UTRs as is likely to improve translational efficiency, and stabilization of 3′-UTRs containing AU-rich elements. Future work will be needed to decipher whether defined phenotypic, biochemical, or molecular sub-groupings of mitochondrial diseases manifest similar or distinctive tissue-specific patterns of transcriptome dysregulation. Indeed, identifying shared mechanisms of pathogenesis across a broad range or targeted subgroup of mitochondrial diseases may facilitate prediction of specific disease cohorts in which a particular therapy is likely to provide greatest benefit.

## Supporting Information

File S1 **Detailed description of data processing and individual bioinformatic analyses.**
(PDF)

File S2 **Excel file of GSEA results of pathway-level transcriptome changes in RC disease subjects’ skeletal muscle and fibroblasts relative to controls.**
(XLS)

File S3 **Thirteen supplemental figures and two supplemental tables.** ***Figure S1*** **. Comparative analysis between our heterogeneous RC disease muscle dataset and public RC disease muscle datasets from three mitochondrial encephalomyopathy subgroups.** Crimi et al. investigated gene expression changes in skeletal muscle biopsies of mitochondrial encephalomyopathy (MEM) subjects organized into 3 subgroups, which included mitochondrial encephalopathy lactic acidosis and stroke (MELAS), progressive external ophthalmoplegia (PEO), and common 4977 bp mitochondrial DNA (mtDNA) deletion subjects [10]. We identified 35 genes that were commonly upregulated in all 3 MEM subgroups, which were also significantly up-regulated in our RC disease muscle group relative to controls by an average of 8.6% (p = 7.0E^−5^). Each box represents the overall expression change of 2 groups of genes in RC disease muscle. The first group is genes commonly upregulated in 3 MEM disease and the second is the genes commonly downregulated. P value conveys the statistical difference between these 2 groups. ***Figure S2*** **. Differential expression of miR132 target genes in RC disease.** 145 genes predicted as miR132 targets by starBase (http://starbase.sysu.edu.cn) were measured in our RC disease data sets. On average, the miR132 target genes were significantly upregulated by an average of 6.5% in RC disease muscle (p = 2.8E^−12^) and downregulated by an average of 1.8% in RC disease FCLs (p = 0.04). A single outlier gene that was found to be upregulated in RC disease FCLs by greater than 50% is Nuclear Factor I/B. ***Figure S3*** **. Target genes of PPAR family TFs were significantly changed on average in primary RC disease.** **(A)** The 142 genes whose promoters contain both TATA and PPARα binding sites were more downregulated in RC disease muscle than genes containing either TATA (p = 1.3×E^−4^) or PPARα (p = 3.1E^−13^) binding sites alone. **(B)** Potential targets of PPAR family TFs were generally downregulated in RC disease muscle and upregulated in RC disease FCLs. All targets have PPRE motif present in their promoters. The Y-axis indicates the average percentage change in RC disease. ***Figure S4*** **. RP genes were concordantly changed in similar studies. (A).** In an independent, public data set downloaded from the NCBI GEO database (GSE10956) that compared human fibroblasts of patients with deficiency of mitochondrial RC complex V (ATP synthase) to controls, the pattern of expression changes in cytosolic ribosomal protein (RP) genes and mitochondrial RP genes was the same we observed in our data set. **(B)** In an independent, public data set downloaded from the NCBI GEO database (GSE5332) that compared mouse embryonic fibroblasts treated with the mTORC1 inhibitor, rapamycin, to controls, mitochondrial RP genes were generally downregulated by rapamycin treatment. The general changes among cytosolic RP genes were insignificant, possibly because the expression of only a few of these genes were measured by the microarray platform used. **(C)** Reanalysis of another public GEO data set (GSE14882) comparing a homogeneous subset of MELAS m.3243A>G patients to controls in a primary tissue (blood) showed that cytosolic and mytochondrial RP genes show different patterns of differential expression in MELAS patients. Cytosolic RP genes were generally upregulated in MELAS while mitochondrial RP genes were neither up- nor downregulated on average. ***Figure S5*** **. Potential binding sites of** ***NRF2*** **and** ***YY1*** **are significantly over-represented within the [-1 kb, +1 kb] promoter regions of both cytosolic and mitochondrial RP genes.** TF binding sites were identified based on the UCSC Genome Browser “*conserved TFBS*” track. ***Figure S6*** **. Concordant differential expression of RP genes. (A).** While mitochondrial RP genes were generally upregulated in RC disease muscle, those mitochondrial RP genes with potential FOXO1 binding sites within their introns were upregulated unanimously and to an even greater degree than were all mitochondrial RP genes as a whole. **(B)** Expression of two forkhead TFs, *FOXK1* and *FOXC2*, showed positive correlation with most cytosolic RP genes (green) across four sample groups (2 tissue types x 2 disease status). However, mitochondrial RP genes (blue) showed much less significant correlation with *FOXK1* and none with *FOXC2* expression. ***Figure S7*** **. Sub-gene level analyses demonstrated differential expression of individual exons in RC disease.** These genes provide examples of representative differential expression patterns within UTRs or exons relative to overall (“gene-level”) expression changes. Each colored box represents a microarray probeset, with shorter boxes at gene ends corresponding to UTRs. Colors indicate direction of expression changes within each gene region in RC disease, where red = upregulated and green = downregulated. ***Figure S8*** **. Comparing the abundance of antisense and sense transcripts showed that antisense transcripts were generally lower than sense transcripts.** Muscle had higher relative antisense transcript expression than FCLs. 5′-UTRs and non-coding (ncRNA) transcripts had higher relative antisense transcript expression than did coding exons and 3′-UTRs. ***Figure S9*** **. The RC disease-control differential expression of sense and antisense was positively correlated in both muscle and FCLs**, indicating that antisense transcripts were usually byproducts of sense transcription. However, a small number of outliers were evident, especially in muscle, which may potentially represent a set of antisense transcripts that are functional. Each dot represents one pair of sense-antisense transcripts, whose size corresponds to their combined fold-change. ***Figure S10*** **. Four representative genes are shown that had detectable antisense transcripts with significantly changed expression in RC disease muscle and FCLs.** Grey shading indicates either data was not available or the antisense transcripts were not detectable. Red and green shading, respectively, indicates a particular sub-gene region was relatively up- or downregulated in RC disease. ***Figure S11*** **. Position-specific differences within the UTRs between control and RC disease data sets.** Normalized data of individual probes are mapped to one percent intervals of UTR probesets and averaged for both control (green) and RC disease (red) groups after data was adjusted for the expression level of the first (for 5′-UTR) or last (for 3′-UTR) exon of the same gene. Lines correspond to the average ratios of each one percent interval and shadows are ranges to indicate the 95% confidence interval, all smoothed by the Lowess (locally weighted scatterplot smoothing) method. In general, UTRs had lower measurements than the corresponding whole gene except for the muscle 5′-UTRs. This may indicate that post-transcriptional regulation via UTRs plays a greater role within the in vivo tissue setting. The control-RC disease differences were prominently amplified when proceeding from the 3′ to 5′ ends within the muscle 3′-UTR, suggesting that different 3′ RNA degradation ratios occur in RC disease. Only effective probesets were used in effort to reduce background noise. ***Figure S12*** **. Relative UTR expression in controls and RC disease.** Ratios and standard deviations were calculated based on the expression measurement of UTR probesets relative to the corresponding expression measurement of the whole gene. Both types of UTRs had higher baseline level in muscle than in FCL (red bars), as is consistent with the likely greater *in vivo* complexity of RNA processing. ***Figure S13.*** **Nutrient-sensing signaling network nodes, mTORC1 and AMPK, have altered activity in pharamacologic-based primary RC dysfunction in human podocytes. (A–E)** Pharmacologic RC inhibition in healthy human podocytes altered mTORC1 and AMPK pathway activities in a time-, glucose- and inhibitor dose-dependent fashion. Human podocyte cells were treated for either **(A)** 16 hours or **(B)** 24 hours with DMEM supplemented with 10% fetal bovine serum in either high glucose (4.5 g/L) or no glucose and either 2 uMol oligomycin (“O”), 2 mMol AICAR (“A”), or 20 nMol rapamycin (“R”). The RC complex V inhibitor, oligomycin, caused reduced phospho-S6 protein expression and increased phospho-AMPK protein expression, particularly in the no glucose condition relative to control cells. As expected, rapamycin reduced phospho-S6 expression and AICAR increased phospho-AMPK expression, regardless of glucose concentration. Interestingly, phospho-AMPK activation was greater with oligomycin than with AICAR. **(C)** Human podocyte cells were treated for 24 hours with 10% fetal bovine serum in high glucose (4.5 g/L) with the RC complex V inhibitor, oligomycin, in concentrations ranging from 0 to 5 uMol. Phospho-S6 expression was reduced with oligomycin ranging from 0.25 to 2 uMol relative to untreated cells. Phospho-AMPK was increased with low (0.25 uMol and 0.5 uMol) oligomycin concentrations relative to untreated cells, but not substantially changed with moderate range (1–2 uMol) oligomycin treatment. **(D–E)** A range of inhibitors that target different RC complexes were used to treat healthy human podocytes in high glucose (4.5 g/L) media at either moderate (**Panel D**) or high (**Panel E**) inhibitor concentrations for short (5 hours) and long (16–24 hour) time exposures. RC complex V was inhibited by oligomycin (“O”) at 2 uMol (**D**) or 5 uMol (**E**). RC complex I was inhibited by rotenone (“Ro”) at 0.1 uMol (**D**) or 0.25 uMol (**E**). RC complex III was inhibited by antimycin A (“AA”) at 0.5 uMol (**D**) or 1 uM (**E**). All three RC inhibitors decreased phospho-S6 expression and increased phospho-AMPK expression, with the greatest effects occurring at higher inhibitor concentrations and longer exposure times. ***Table S1*** **. Comparison of our RC disease muscle transcriptome data with that of a prior reported mitochondrial encephalomyopathy muscle transcriptome data set.** 6 genes were commonly up-regulated in muscle biopsy array data from 4 different mitochondrial diseases, upon our reanalysis of the same public array data as detailed in **Fig. S1 in File S3** [10]. Numbers convey percent increased expression. MELAS, mitochondrial encephalomyopathy, lactic acidosis, and stroke-like episodes. PEO, progressive external ophthalmoplegia. mtDNA, mitochondrial DNA. RC, respiratory chain. ***Table S2*** **.** **List of differentially expressed genes (DEGs) that were concordantly changed in RC disease patient muscle and FCLs relative to controls.** Red and green font indicates genes that were, respectively, up- and downregulated in RC disease.
(PDF)
