# Supplementary material for: Primary Respiratory Chain Disease Causes Tissue-Specific Dysregulation of the Global Transcriptome and Nutrient-Sensing Signaling Network
Source: PLoS One. 2013 Jul 24;8(7):e69282. doi: 10.1371/journal.pone.0069282 (PMC3722174; doi:10.1371/journal.pone.0069282)
Supplement: File S1 — Detailed description of data processing and individual bioinformatic analyses. (PDF) [file pone.0069282.s001.pdf]

Microarray Data Processing

Remapping probes

The Affymetrix Human Exon 1.0ST microarrays include 6,656,400 features, of which the company provides the 25mer probe sequences of 5,431,924. We downloaded those sequences from Affymetrix website and re-mapped them to human reference genome hg19. A probe filtering step then removed from further processing 435,624 probes that mapped to multiple loci, had no perfect match, or included any sequence variations according to dbSNP build 132.

Assign probes to probesets and genes

The design of the Exon 1.0ST platform includes a large portion of probes mapped to putative genes or genes with unknown functions. For our gene-level analysis, we only wanted to investigate expression changes of known genes. We then downloaded the RefSeq track from UCSC Genome Browser and assigned probes to sub-gene regions based on their re-mapped locations. Following the concept defined by Affymetrix, we call the regions PSR (probe selection regions), which are non-overlapping segments within the same gene. Probes assigned to the same PSR constitute a probeset. There are four types of probesets based on location: exon, 5'-UTR, 3'-UTR, and junction probesets. The last probeset type is composed of probes that map to exon-intron junctions and usually include a single probe. Probesets located within both exon and UTR are treated as exon probesets. In summary, 1,359,659 unique probes were assigned to 246,604 probesets. The average number of probes per probeset is 5.57 and 91.2% of the probesets include at least 3 probes.

We further assigned probesets to NCBI genes by mapping RefSeq and NCBI gene IDs. There were 20,741 unique genes that included at least one probeset, among which 20,518 genes included at least one exon probeset.

Raw data processing

Muscle and FCL data were processed separately. After retrieving raw data from the CEL file, we normalized the log2-transformed probe-level measurements with the Loess method. The muscle samples included three pairs of technical replicates, for which we simply took the average of replicates after normalization to get a single measurement of each gene from each subject. The normalized data were summarized into probeset-level

data using Li-Wong's algorithm. UTR and exon probesets were used to get gene-level measurements also using Li-Wong's method. Both probeset-level and gene-level data were normalized again using the Loess method.

Selection of effective probesets

Probesets measuring silenced transcripts, such as most antisense probesets, would only generate background noise. For analyses requiring high specificity or involving mostly silenced transcripts, we used a only a subset of probesets that were called effective. An effective probeset satisfies either of these two conditions:

1. The probeset includes three or more probes and has significant average probe-probe correlation ($p < 0.05$) since probes of the same probeset measure the same target. The significance is based on Z-transformation of correlation coefficient. The figure on the right shows the distribution of average probe-probe correlations of all probesets.
2. The probeset is called 'present' in at least three of the 20 samples. The probe-level measurements of a 'present' probeset should be significantly higher than background ($p < 0.05$) according to t test. The background is composed of all probes not mapped to any RefSeq gene.

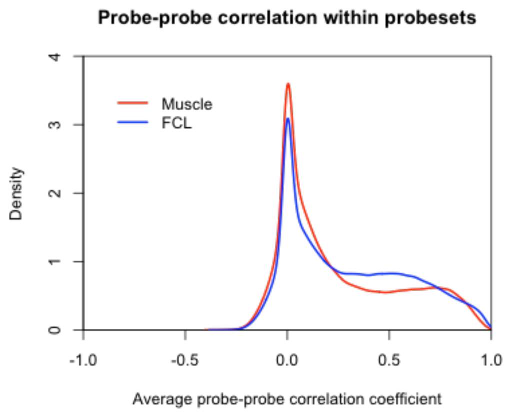

As a result, 134,603/5,508 and 162,726/9,083 sense/antisense probesets were effective in muscle and FCL respectively, and 124,185/4,772 of them were common to both tissues.

Statistical analysis of differential expression

Gene-level analysis

We utilized PCA for unsupervised sample clustering, using all autosomal genes. The first two principal components were unable to clearly distinguish sample groups.

DE of all 20,741 unique genes between 8 control samples and 12 mitochondrial RC disease samples were calculated with the SAM method for both tissues. Using $p = 0.05$ as cutoff, we selected up- and downregulated genes from both tissues to obtain four gene lists. We further selected sub-lists, such as genes upregulated in

both tissue types, to perform functional analysis using the DAVID web tool. The gene categories over-represented by each gene list were summarized.

TFBS analysis

Locations of human/mouse/rat conserved TF binding sites were downloaded from the UCSC genome browser (The *conserved TFBS* track). Sites were mapped to RefSeq genes by *findOverlaps* function from *Biostrings* package. A gene would be called as a potential target of a TF if it had a binding site of that TF within its [-1 kb, 1 kb] regions around TSS or intron. Introns larger than 200 kb were excluded because they were likely to contain many binding sites.

The 15 bp PWM of PPRE (peroxisome proliferator response element) was downloaded from the literature [1] and mapped to hg19 using the *matchPWM* function from *Biostrings* package for sites with at least 95% similarity. Fewer than 2,000 sites were identified due to the high stringency, but they were enriched around promoter regions. We identified 261 potential target genes whose [-10 kb, 1 kb] promoter included at least one matching site. Differential expression of these genes was compared to non-target genes (see **Figure S3B**), which showed that they were generally downregulated in RC disease muscle and upregulated in RC disease FCLs.

RP gene analysis

We identified 60 cytosolic and 75 mitochondrial ribosomal protein genes that were measured by at least one probeset. These genes all encoded proteins constituting ribosomal subunits, but do not include pseudogenes or genes regulating ribosome biogenesis, such as S6 kinase genes.

UTR and exon analysis

We applied the SAM method to get a p value for each sense probeset. We considered a gene having a sub-gene event, such as alternative splicing or faster degradation, if it satisfied both conditions below:

1. The probeset itself was significantly changed. For each type of sub-gene region of each gene, we selected the probeset with the smallest p value and adjusted the p value with Bonferroni correction (p multiplied by number of probesets). The adjusted p value had to be < 0.05 to be called a sub-gene event.
2. The direction of change at the probeset-level was opposite to the direction of change at the gene-level, OR the probeset-level p value was significantly smaller than the gene-level p value with a Z score difference

greater than 3. Z = (T1-T0)/sqrt($SE1^2$+$SE2^2$), where T1 and T0 are probeset and gene t statistics, respectively, and SE1 and SE2 are the corresponding standard errors (estimated assuming unequal variance).

3'-UTR and AU-rich elements

We performed the following analyses to investigate the association between the presence of AU-rich elements (AREs) within 3'-UTRs and the differential expression of 3'-UTRs.

The correlation coefficients of genes to each RP gene were calculated by the meta-analysis of four sample groups (two cell types times two disease groups), using the *metacor.DSL* function of the *metacor* package.

UTR and exon analysis

AREsite is an online resource (http://rna.tbi.univie.ac.at/cgi-bin/AREsite.cgi) that provides lists of genes having AREs within their 3'-UTR based upon phylogenetic conservation and structural context of those sequences [2]. We downloaded from this site four lists of genes including AREs motifs: AUUUA, WWAUUUAWW, WWWAUUUAWWW, and WWWWAUUUAWWWW. Since the latter ones are inclusive of the earlier ones, we filtered the lists to make them exclusive of each other. That is, the list of AUUUA would not include any genes within the list of WWAUUUAWW and so forth. AREsite annotates genes with Ensemble gene IDs, and we matched them to NCBI gene IDs via Bioconductor package "org.Hs.eg.db". Consequently, the final gene lists included 5,423/ 2,428/ 1,712/ 2,632 unique genes, respectively, for each of the for groups.

To apply the AREsite gene lists to our microarray data, we limited the analysis only to effective probesets to improve specificity. There were 7,774 and 9,764 effective 3'-UTR probesets in muscle and FCLs, respectively. We calculated the group difference of these probesets, which is equal to the log2-ratio of group means, and took the average of the probesets within the same genes. As a result, we obtained the 3'-UTR differential expression of 7,353 and 9,065 unique genes, respectively. We assigned these genes to any of the four AREsite gene groups or a "NONE" group of genes having no 3'-UTR ARE. We then compared the average differential expression of the five groups and concluded that there was a strong association between differential expression of 3'-UTRs and the presence of AREs.

We also observed that 3'-UTRs having AREs tended to be longer than 3'-UTR without AREs. To exclude the possibility that the observed association between ARE and differential expression was caused by 3'-UTR length,

we performed an analysis to compare the average differential expression of non-ARE 3'-UTRs longer than 1 kb (n = 486) and ARE-including 3'-UTRs shorter than 1 kb (n = 1,661) in muscle. The average expression change was +2.2% versus +9.2% ($p = 1.2e^{-20}$). Therefore, short 3'-UTRs including AREs were more changed than were longer 3'-UTRs without AREs, where the association was independent of 3'-UTR length. We also performed a similar analysis using ARE-containing gene lists downloaded from ARED (http://brp.kfshrc.edu.sa/ARED) and obtained similar, but less pronounced results.

Antisense transcripts

Probes completely mapped to antisense transcripts were grouped into antisense probesets. Only effective probesets that included at least three probes were used for the analysis. Differential expression of antisense probesets between controls and RC disease subjects was calculated using the SAM method.

Re-analysis of GEO public data sets

If the data set was generated from an Affymetrix platform, we downloaded the raw CEL files from the NCBI GEO database and processed them using the custom library file provided by the BRAINARRAY project [3]. These were normalized and summarized into gene-level data by the RMA method. If the data set was generated from platforms from other vendors, we directly downloaded the processed data from NCBI GEO and mapped the original gene identifiers to NCBI Entrez gene IDs.

References for File S1

1. Lemay DG, Hwang DH (2006) Genome-wide identification of peroxisome proliferator response elements using integrated computational genomics. J Lipid Res 47: 1583-1587.
2. Gruber AR, Fallmann J, Kratochvill F, Kovarik P, Hofacker IL (2011) AREsite: a database for the comprehensive investigation of AU-rich elements. Nucleic Acids Res 39: D66-69.
3. Dai M, Wang P, Boyd AD, Kostov G, Athey B, et al. (2005) Evolving gene/transcript definitions significantly alter the interpretation of GeneChip data. Nucleic Acids Res 33: e175.
