# Supplementary material for: Primary Respiratory Chain Disease Causes Tissue-Specific Dysregulation of the Global Transcriptome and Nutrient-Sensing Signaling Network
Source: PLoS One. 2013 Jul 24;8(7):e69282. doi: 10.1371/journal.pone.0069282 (PMC3722174; doi:10.1371/journal.pone.0069282)
Supplement: File S3 — Thirteen supplemental figures and two supplemental tables. Figure S1 . Comparative analysis between our heterogeneous RC disease muscle dataset and public RC disease muscle datasets from three mitochondrial encephalomyopathy subgroups. Crimi et al. investigated gene expression changes in skeletal muscle biopsies of mitochondrial encephalomyopathy (MEM) subjects organized into 3 subgroups, which included mitochondrial encephalopathy lactic acidosis and stroke (MELAS), progressive external ophthalmoplegia (PEO), and common 4977 bp mitochondrial DNA (mtDNA) deletion subjects [10]. We identified 35 genes that were commonly upregulated in all 3 MEM subgroups, which were also significantly up-regulated in our RC disease muscle group relative to controls by an average of 8.6% (p = 7.0E−5). Each box represents the overall expression change of 2 groups of genes in RC disease muscle. The first group is genes commonly upregulated in 3 MEM disease and the second is the genes commonly downregulated. P value conveys the statistical difference between these 2 groups. Figure S2 . Differential expression of miR132 target genes in RC disease. 145 genes predicted as miR132 targets by starBase (http://starbase.sysu.edu.cn) were measured in our RC disease data sets. On average, the miR132 target genes were significantly upregulated by an average of 6.5% in RC disease muscle (p = 2.8E−12) and downregulated by an average of 1.8% in RC disease FCLs (p = 0.04). A single outlier gene that was found to be upregulated in RC disease FCLs by greater than 50% is Nuclear Factor I/B. Figure S3 . Target genes of PPAR family TFs were significantly changed on average in primary RC disease. (A) The 142 genes whose promoters contain both TATA and PPARα binding sites were more downregulated in RC disease muscle than genes containing either TATA (p = 1.3×E−4) or PPARα (p = 3.1E−13) binding sites alone. (B) Potential targets of PPAR family TFs were generally downregulated in RC disease muscle and upregulat [file pone.0069282.s003.pdf]

Figure S1

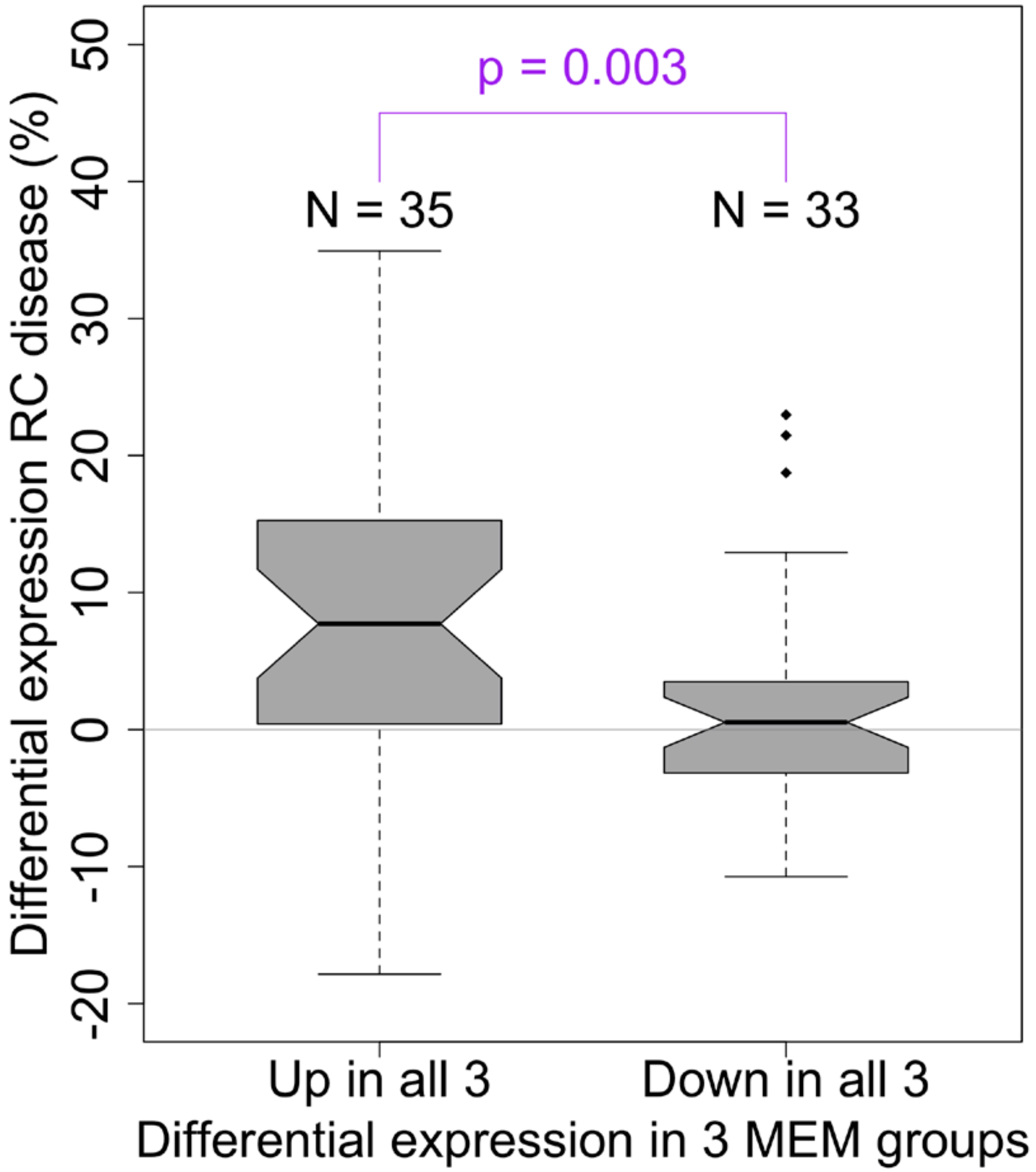
p = 0.003
N = 35
N = 33
Differential expression RC disease (%)
50
40
30
20
10
0
-10
-20
Up in all 3
Down in all 3
Differential expression in 3 MEM groups

Figure S2

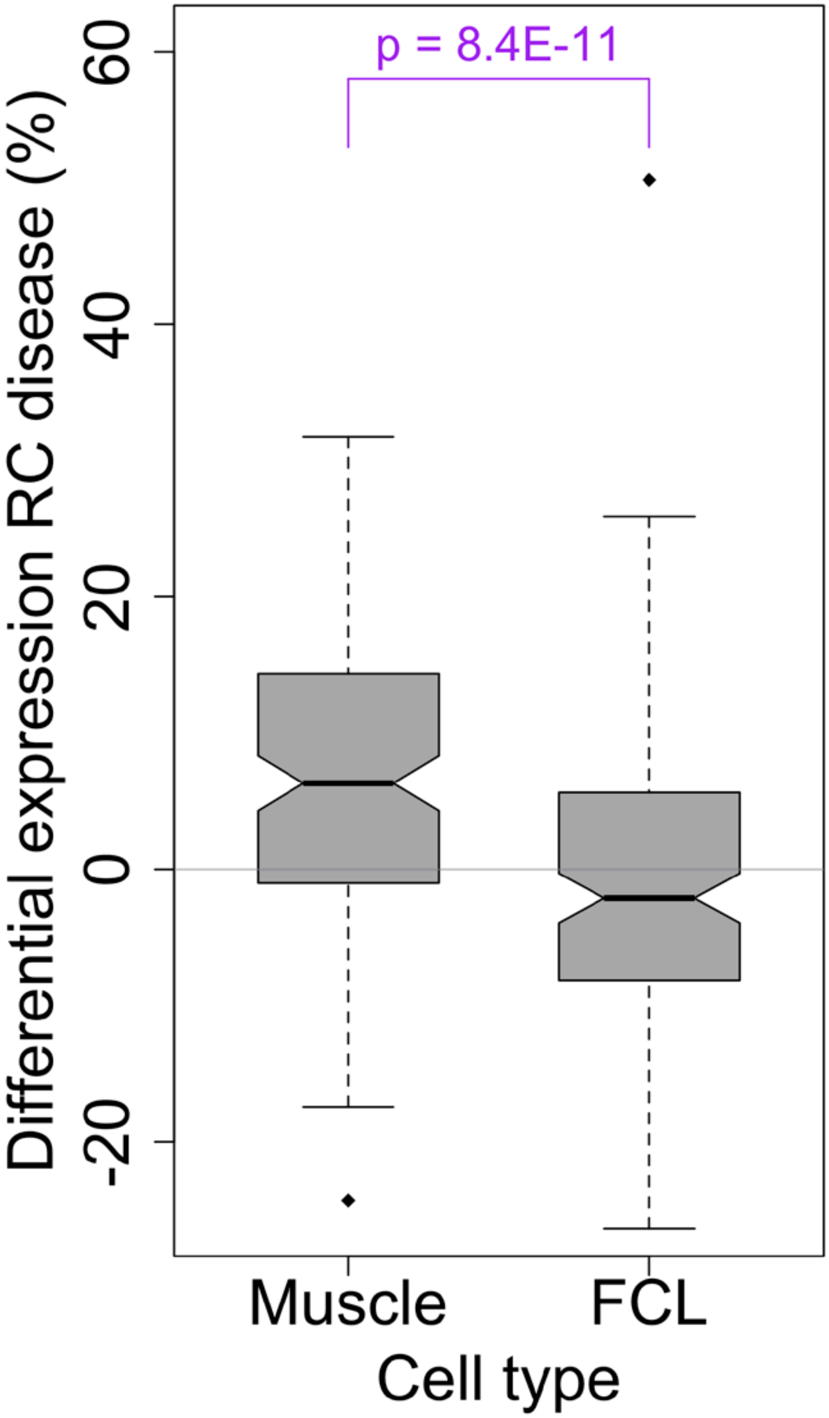
p = 8.4E-11
Differential expression RC disease (%)
60
40
20
0
-20
Muscle
FCL
Cell type

Figure S3

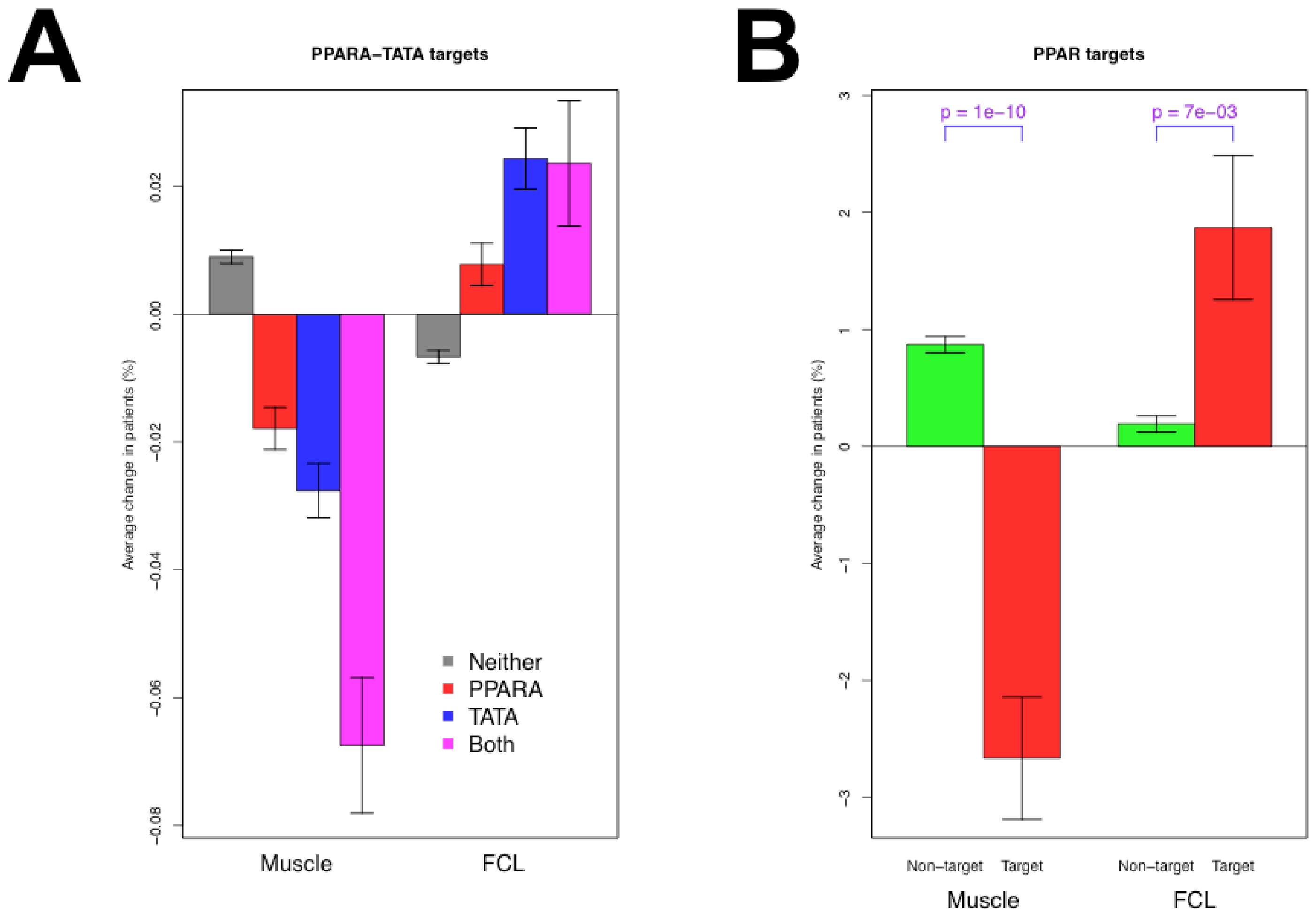
A
PPARA–TATA targets
Average change in patients (%)
0.02
0.00
−0.02
−0.04
−0.06
−0.08
Neither
PPARA
TATA
Both
Muscle
FCL
B
PPAR targets
Average change in patients (%)
3
2
1
0
−1
−2
−3
p = 1e−10
p = 7e−03
Non−target
Target
Non−target
Target
Muscle
FCL

Figure S4

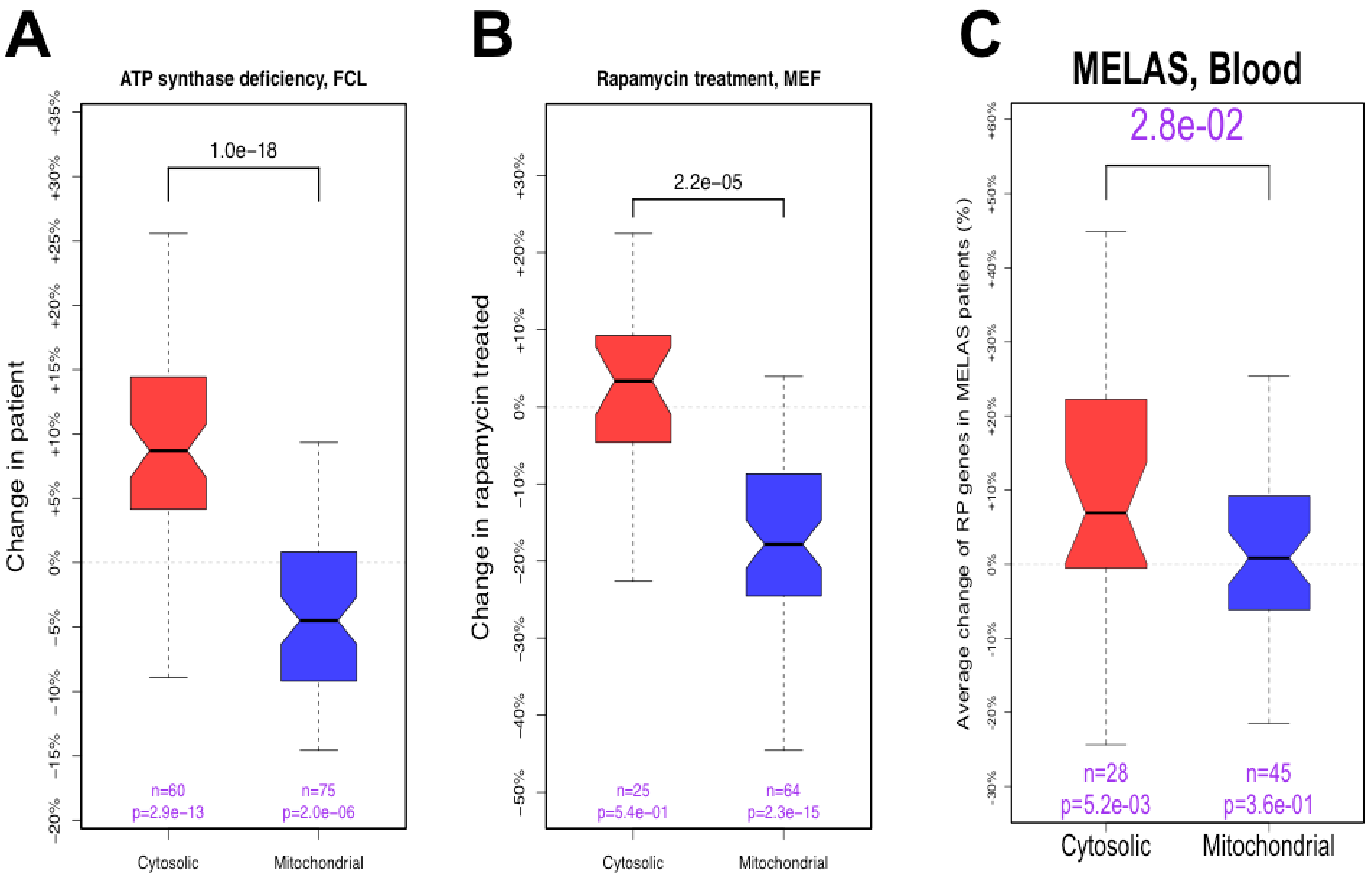
A
ATP synthase deficiency, FCL
1.0e−18
Change in patient
n=60
p=2.9e−13
n=75
p=2.0e−06
Cytosolic
Mitochondrial
B
Rapamycin treatment, MEF
2.2e−05
Change in rapamycin treated
n=25
p=5.4e−01
n=64
p=2.3e−15
Cytosolic
Mitochondrial
C
MELAS, Blood
2.8e-02
Average change of RP genes in MELAS patients (%)
n=28
p=5.2e-03
n=45
p=3.6e-01
Cytosolic
Mitochondrial

Figure S5

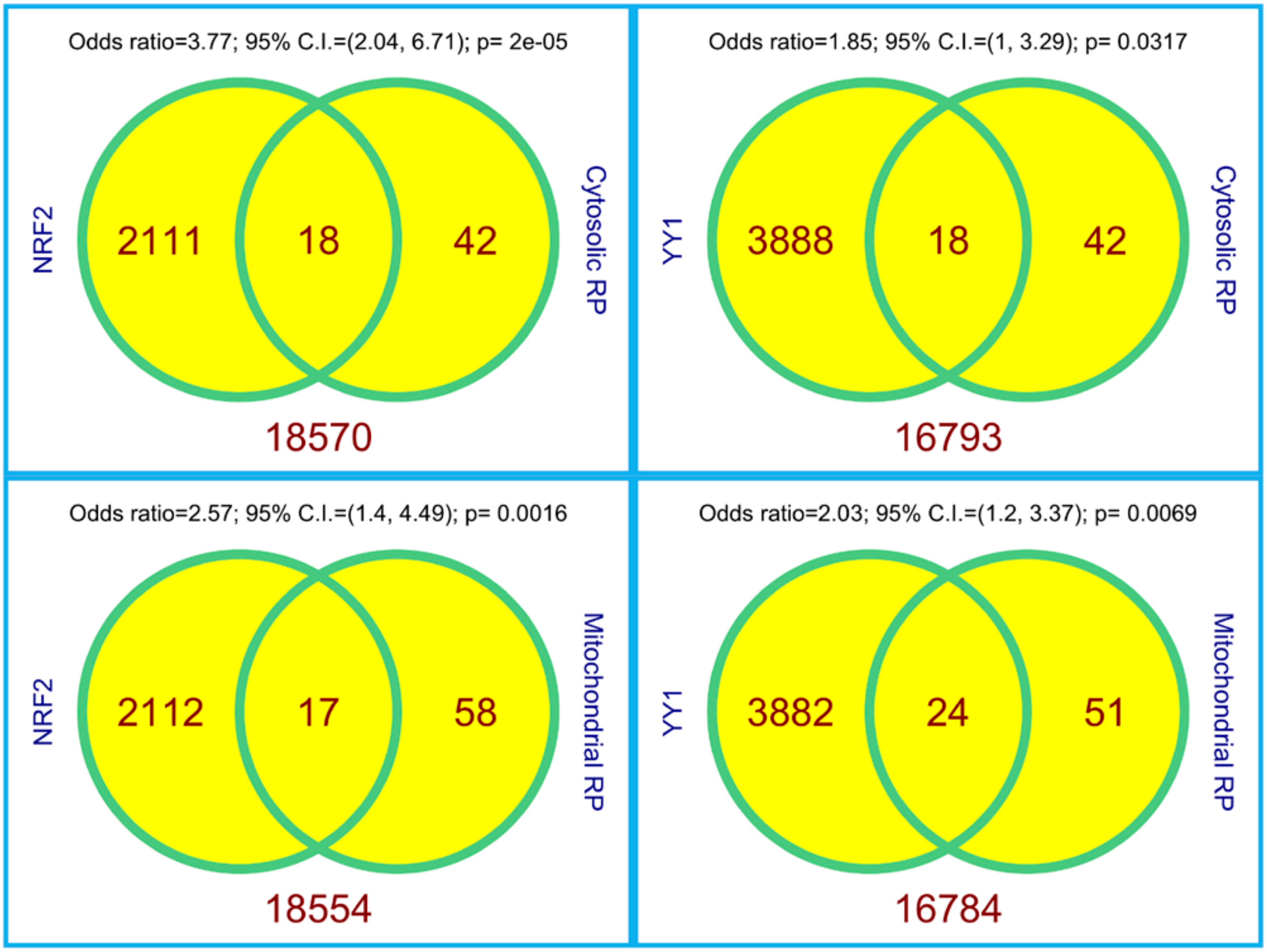
Odds ratio=3.77; 95% C.I.=(2.04, 6.71); p= 2e-05
NRF2
2111
18
42
Cytosolic RP
18570
Odds ratio=1.85; 95% C.I.=(1, 3.29); p= 0.0317
YY1
3888
18
42
Cytosolic RP
16793
Odds ratio=2.57; 95% C.I.=(1.4, 4.49); p= 0.0016
NRF2
2112
17
58
Mitochondrial RP
18554
Odds ratio=2.03; 95% C.I.=(1.2, 3.37); p= 0.0069
YY1
3882
24
51
Mitochondrial RP
16784

Figure S6

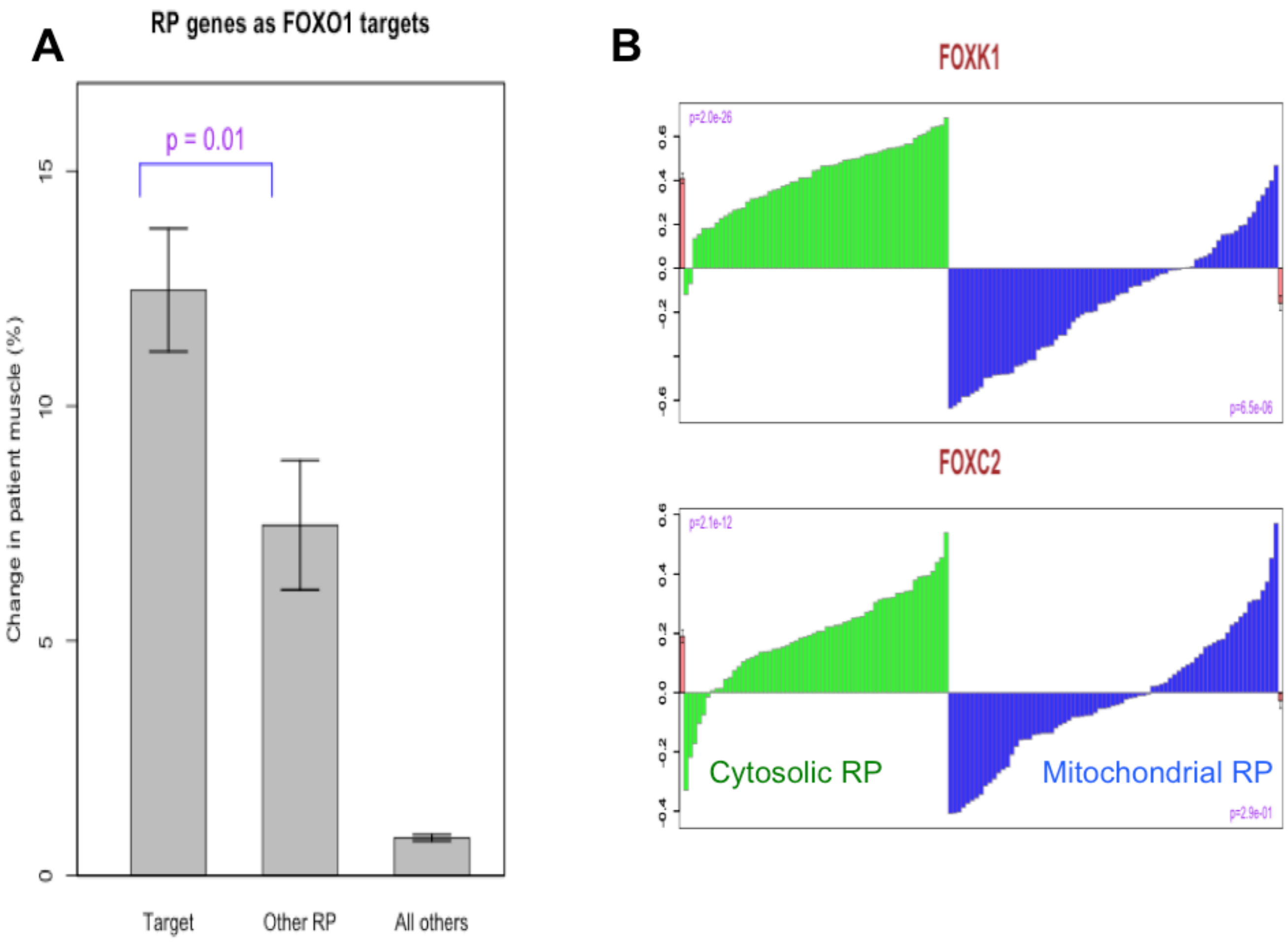
A
RP genes as FOXO1 targets
p = 0.01
Change in patient muscle (%)
0
5
10
15
Target
Other RP
All others
B
FOXK1
p=2.0e-26
p=6.5e-06
FOXC2
p=2.1e-12
p=2.9e-01
Cytosolic RP
Mitochondrial RP

Figure S7

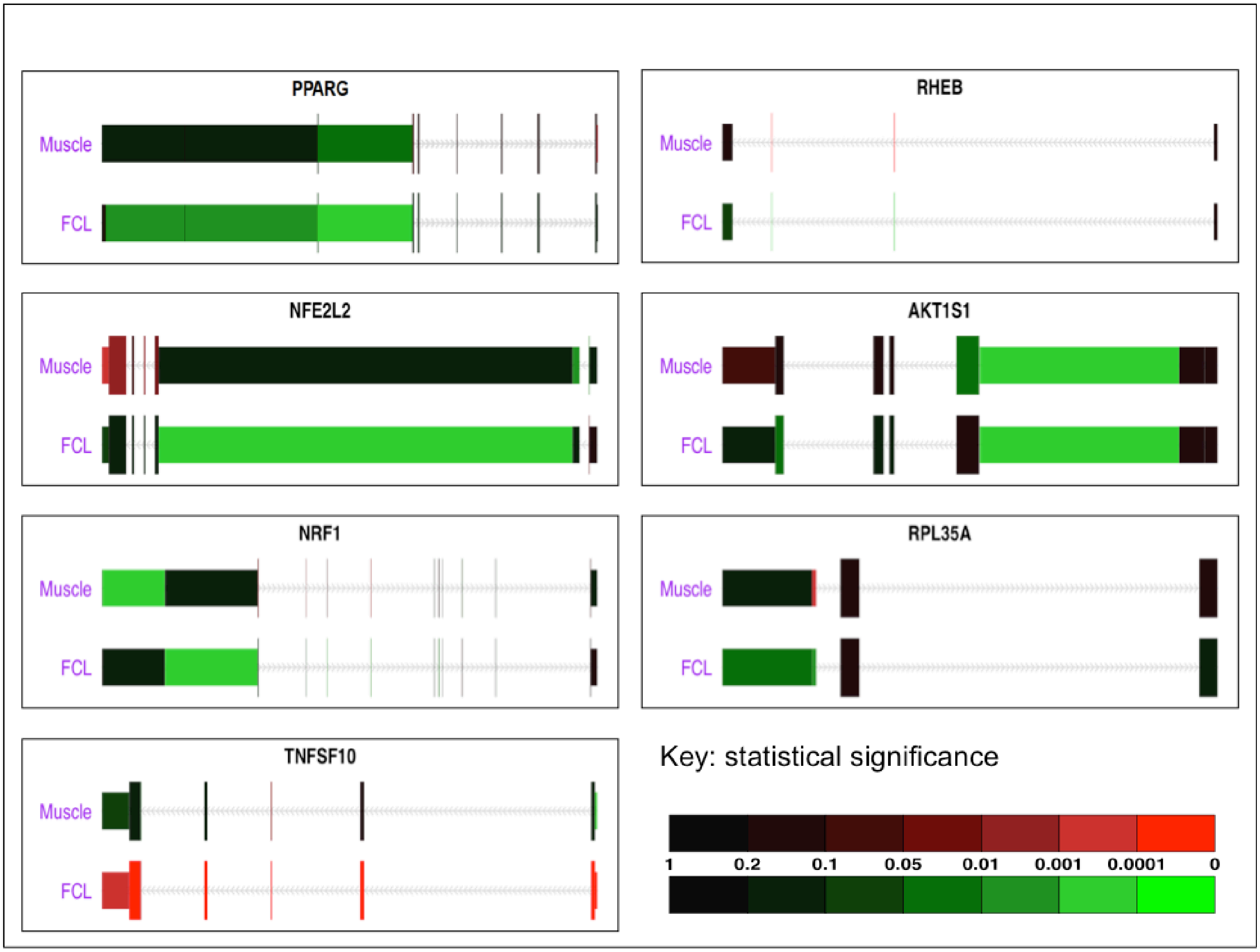
PPARG
Muscle
FCL
RHEB
Muscle
FCL
NFE2L2
Muscle
FCL
AKT1S1
Muscle
FCL
NRF1
Muscle
FCL
RPL35A
Muscle
FCL
TNFSF10
Muscle
FCL
Key: statistical significance
1
0.2
0.1
0.05
0.01
0.001
0.0001
0

Figure S8

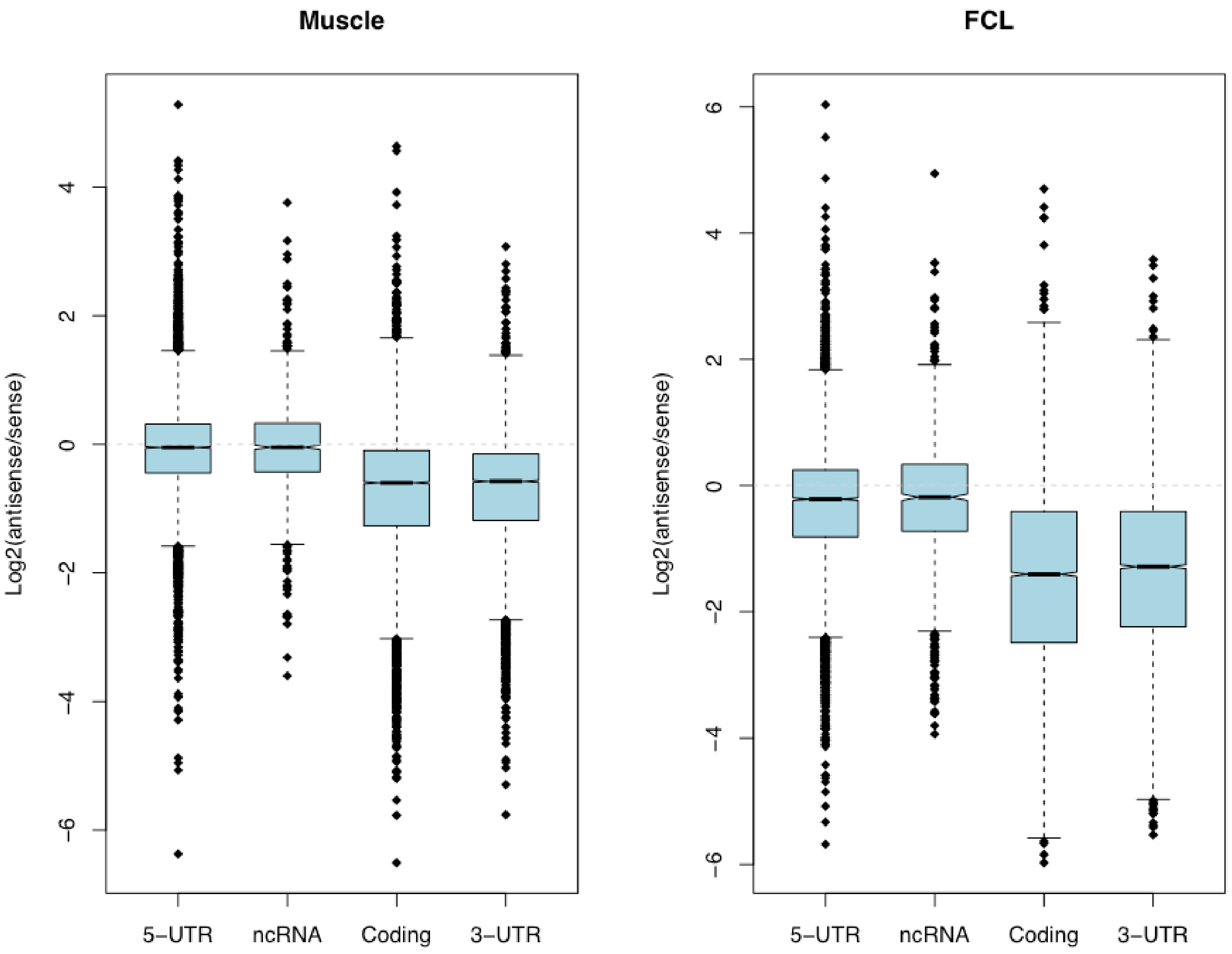

Muscle
FCL
Log2(antisense/sense)
Log2(antisense/sense)
5−UTR
ncRNA
Coding
3−UTR
5−UTR
ncRNA
Coding
3−UTR
4
2
0
−2
−4
−6
6
4
2
0
−2
−4
−6

Figure S9

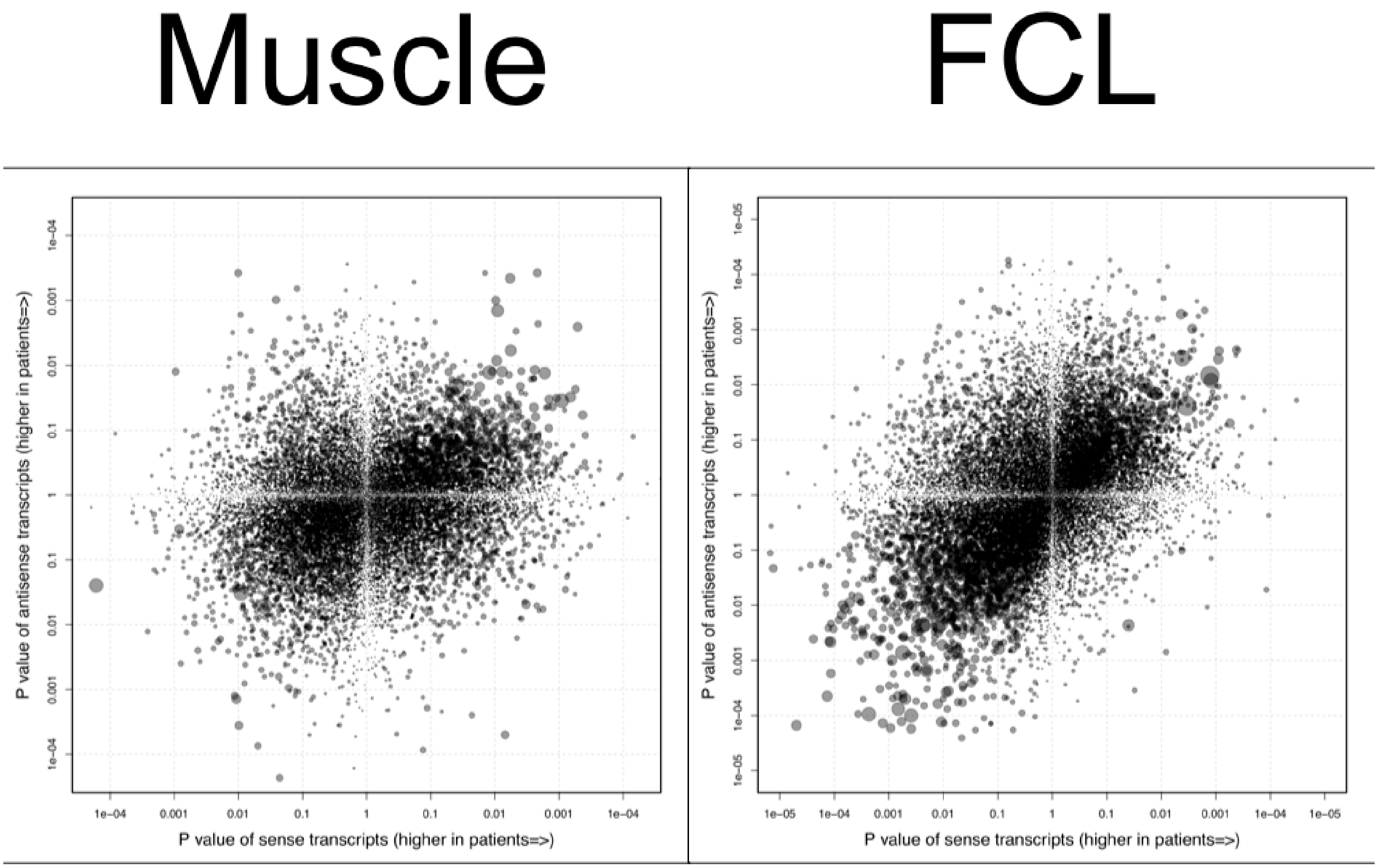
Muscle
FCL
P value of antisense transcripts (higher in patients=>)
P value of sense transcripts (higher in patients=>)
P value of antisense transcripts (higher in patients=>)
P value of sense transcripts (higher in patients=>)

Figure S10

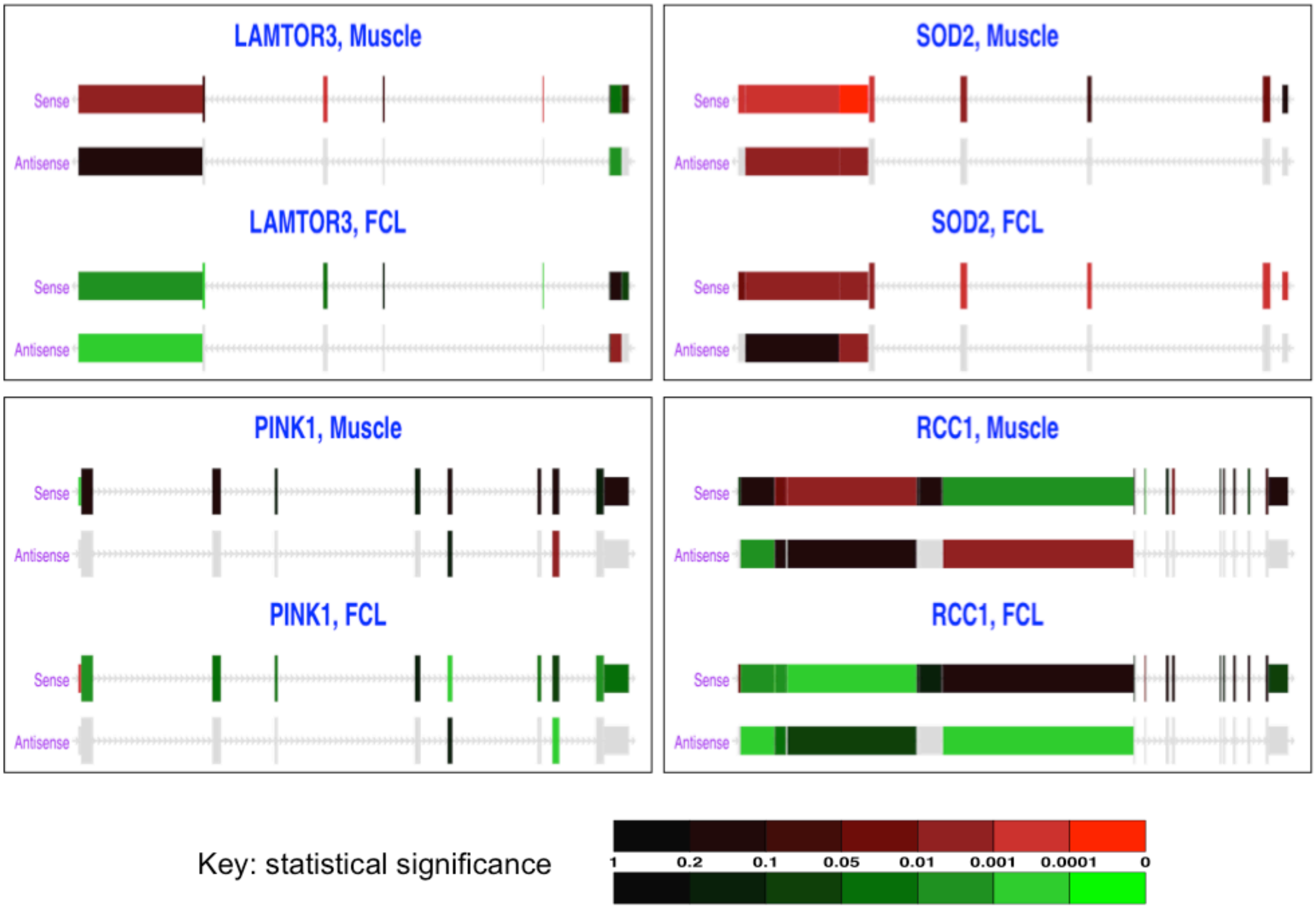
LAMTOR3, Muscle
Sense
Antisense
LAMTOR3, FCL
Sense
Antisense
SOD2, Muscle
Sense
Antisense
SOD2, FCL
Sense
Antisense
PINK1, Muscle
Sense
Antisense
PINK1, FCL
Sense
Antisense
RCC1, Muscle
Sense
Antisense
RCC1, FCL
Sense
Antisense
Key: statistical significance
1
0.2
0.1
0.05
0.01
0.001
0.0001
0

Figure S11

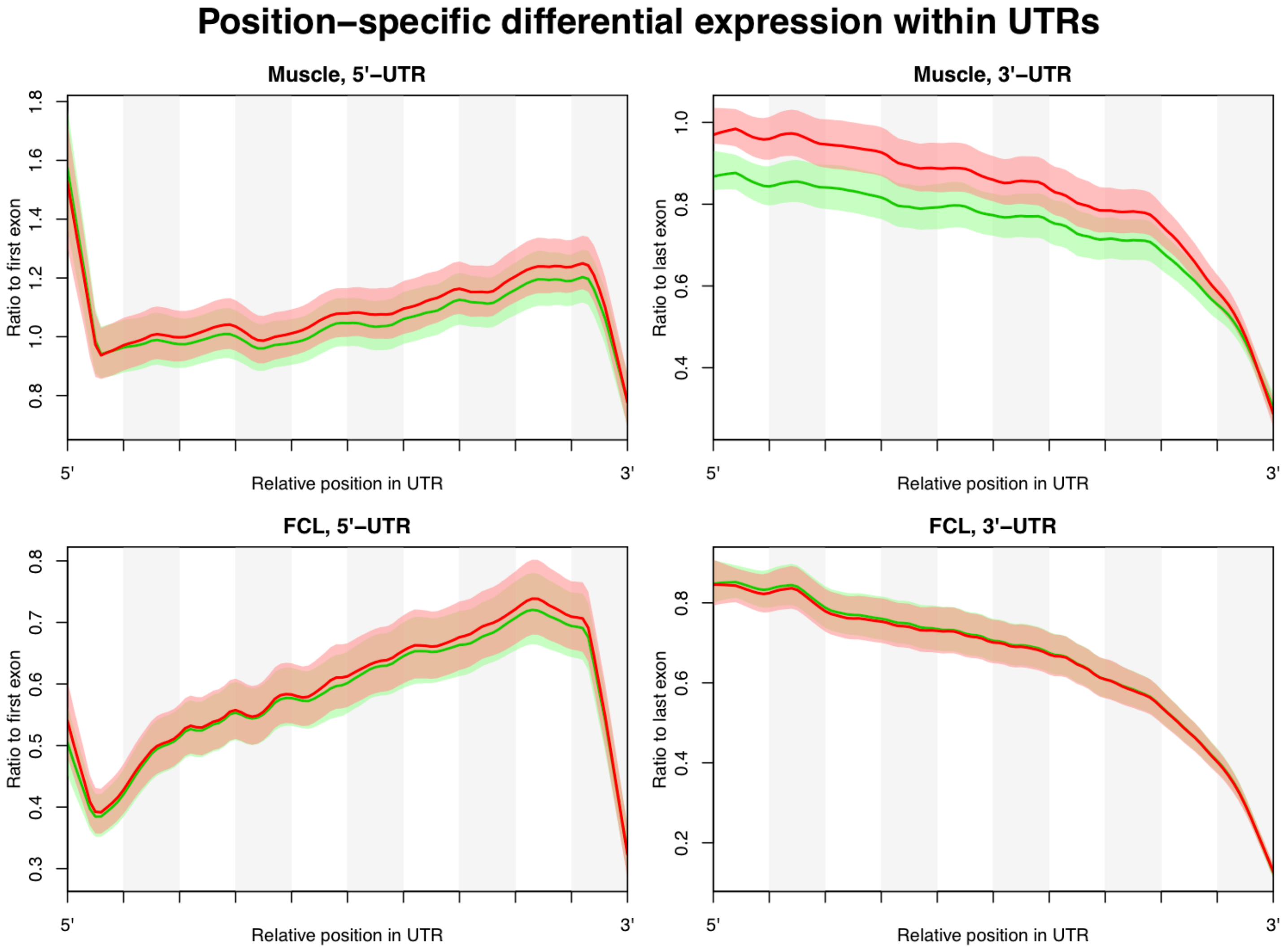

Position–specific differential expression within UTRs
Muscle, 5'–UTR
Muscle, 3'–UTR
FCL, 5'–UTR
FCL, 3'–UTR
Ratio to first exon
Ratio to last exon
Relative position in UTR
5'
3'
0.8
1.0
1.2
1.4
1.6
1.8
0.4
0.6
0.8
1.0
0.3
0.4
0.5
0.6
0.7
0.8
0.2
0.4
0.6
0.8

Figure S12

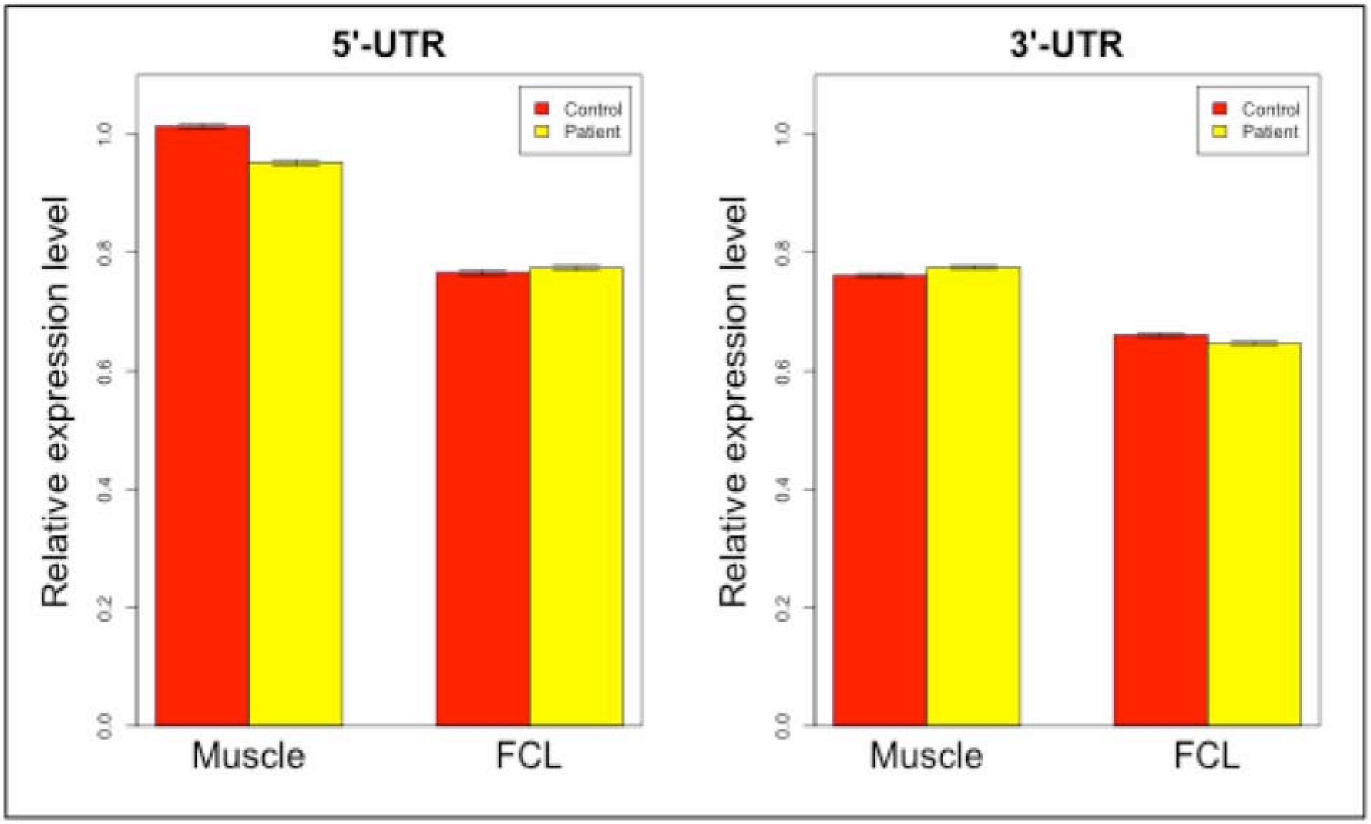
5'-UTR
3'-UTR
Control
Patient
Relative expression level
0.0
0.2
0.4
0.6
0.8
1.0
Muscle
FCL

Figure S13

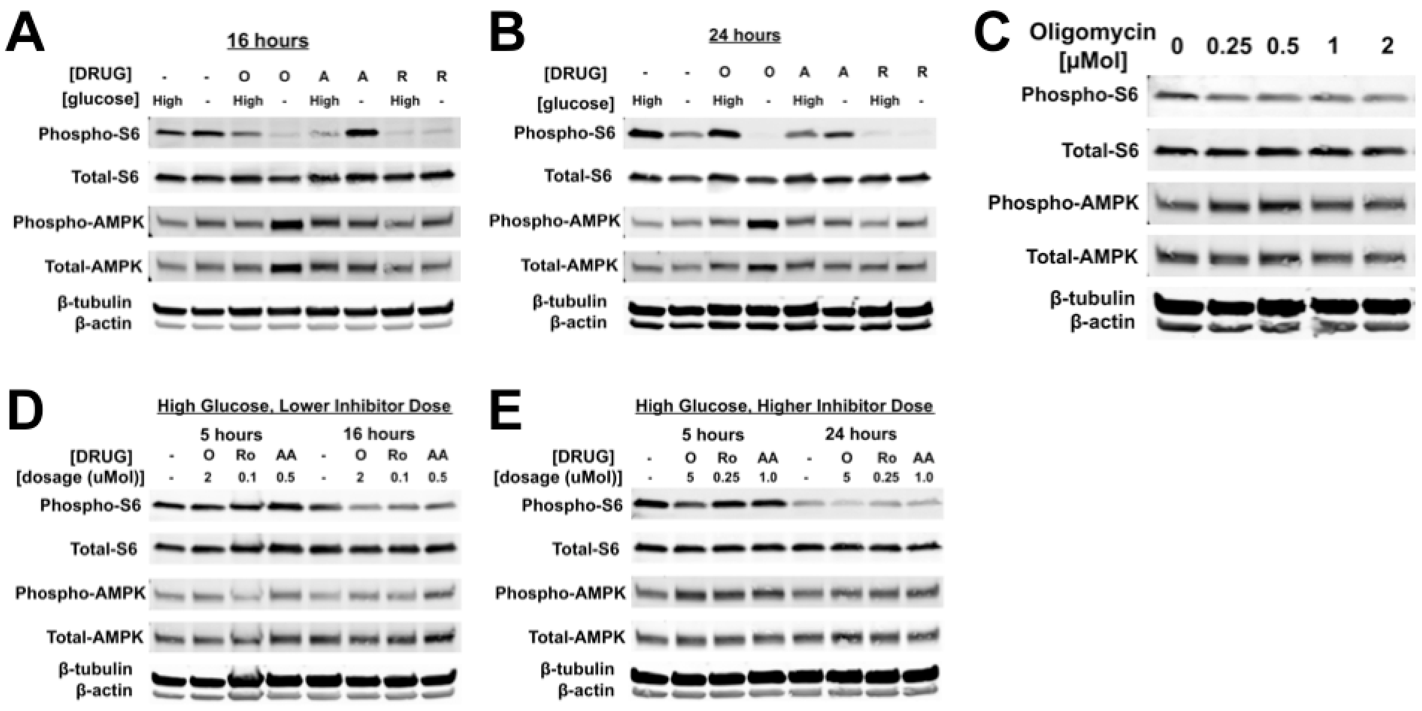
A
16 hours
[DRUG] - - O O A A R R
[glucose] High - High - High - High -
Phospho-S6
Total-S6
Phospho-AMPK
Total-AMPK
β-tubulin
β-actin
B
24 hours
[DRUG] - - O O A A R R
[glucose] High - High - High - High -
Phospho-S6
Total-S6
Phospho-AMPK
Total-AMPK
β-tubulin
β-actin
C
Oligomycin [μMol] 0 0.25 0.5 1 2
Phospho-S6
Total-S6
Phospho-AMPK
Total-AMPK
β-tubulin
β-actin
D
High Glucose, Lower Inhibitor Dose
5 hours
16 hours
[DRUG] - O Ro AA - O Ro AA
[dosage (uMol)] - 2 0.1 0.5 - 2 0.1 0.5
Phospho-S6
Total-S6
Phospho-AMPK
Total-AMPK
β-tubulin
β-actin
E
High Glucose, Higher Inhibitor Dose
5 hours
24 hours
[DRUG] - O Ro AA - O Ro AA
[dosage (uMol)] - 5 0.25 1.0 - 5 0.25 1.0
Phospho-S6
Total-S6
Phospho-AMPK
Total-AMPK
β-tubulin
β-actin

Zhang et al_ File S3

Table S1.

**Gene**	**MELAS**	**PEO**	**mtDNA deletion**	**RC disease**	**GENE NAME**
PGRMC2	28.35	13.82	27.43	13.96	progesterone receptor membrane component 2
UBXN2B	28.86	21.15	22.23	14.89	UBX domain protein 2B
ESYT1	46.67	63.96	37.53	28.61	extended synaptotagmin-like protein 1
DNAJC15	65.72	26.43	42.86	26.89	DnaJ (Hsp40) homolog, subfamily C, member 15
EXOC2	23.64	30.96	20.15	21.43	exocyst complex component 2
SPCS3	10.75	12.11	10.36	32.90	signal peptidase complex subunit 3 homolog (*S. cerevisiae*)

Zhang et al_ File S3

Table S2

ID	Symbol	Muscle Mean_Ctrl	Muscle Mean_Mito	Muscle Mito-Ctrl	Muscle Fold_change	Muscle p_SAM	Muscle FDR_SAM	FCL Mean_Ctrl	FCL Mean_Mito	FCL Mito-Ctrl	FCL Fold_change	FCL p_SAM	FCL FDR_SAM	Full name
6648	SOD2	9.92	10.44	0.52	1.43	0.01	0.04	10.75	11.56	0.80	1.75	0.00	0.08	superoxide dismutase 2, mitochondrial
30001	ERO1L	6.75	7.17	0.42	1.34	0.01	0.04	10.34	10.56	0.22	1.16	0.04	0.16	ERO1-like (S. cerevisiae)
29103	DNAJC15	7.33	7.68	0.34	1.27	0.02	0.06	9.77	10.10	0.33	1.26	0.02	0.10	DnaJ (Hsp40) homolog, subfamily C, member 15
81671	VMP1	7.87	8.19	0.32	1.25	0.01	0.04	10.69	10.83	0.14	1.10	0.03	0.14	vacuole membrane protein 1
3454	IFNAR1	7.39	7.65	0.27	1.20	0.04	0.11	9.66	9.90	0.25	1.19	0.04	0.14	interferon (alpha, beta and omega) receptor 1
25853	DCAF12	7.42	7.67	0.25	1.19	0.03	0.09	9.09	9.27	0.18	1.13	0.02	0.12	DDB1 and CUL4 associated factor 12
1317	SLC31A1	6.61	6.85	0.24	1.18	0.02	0.05	10.61	10.84	0.23	1.17	0.02	0.10	solute carrier family 31 (copper transporters), member 1
79607	FAM118B	7.30	7.53	0.23	1.18	0.03	0.06	9.23	9.37	0.15	1.11	0.01	0.10	family with sequence similarity 118, member B
54994	C20orf11	7.39	7.62	0.23	1.17	0.01	0.04	8.92	9.02	0.10	1.07	0.03	0.12	chromosome 20 open reading frame 11
5663	PSEN1	7.11	7.34	0.22	1.17	0.03	0.09	9.76	9.97	0.21	1.15	0.01	0.08	presenilin 1
3920	LAMP2	8.49	8.71	0.22	1.17	0.04	0.11	11.09	11.26	0.16	1.12	0.03	0.12	lysosomal-associated membrane protein 2
54877	ZCCHC2	6.85	7.06	0.21	1.16	0.05	0.12	8.29	8.53	0.23	1.17	0.03	0.14	zinc finger, CCHC domain containing 2
2120	ETV6	7.01	7.22	0.21	1.16	0.04	0.11	9.33	9.50	0.18	1.13	0.03	0.12	ets variant 6
55731	C17orf63	7.43	7.63	0.20	1.15	0.01	0.04	8.33	8.43	0.10	1.07	0.03	0.12	chromosome 17 open reading frame 63
55084	SOBP	7.15	7.34	0.19	1.14	0.01	0.04	7.00	7.21	0.21	1.16	0.05	0.16	sine oculis binding protein homolog (Drosophila)
4087	SMAD2	7.81	8.00	0.19	1.14	0.01	0.03	9.57	9.70	0.14	1.10	0.02	0.12	SMAD family member 2
84945	ABHD13	7.34	7.52	0.18	1.13	0.03	0.06	8.96	9.13	0.17	1.13	0.03	0.12	abhydrolase domain containing 13
284058	KIAA1267	7.23	7.40	0.17	1.12	0.03	0.09	8.43	8.62	0.18	1.13	0.04	0.16	KIAA1267
56160	NDNL2	6.88	7.02	0.14	1.10	0.02	0.05	8.17	8.29	0.12	1.09	0.03	0.14	necdin-like 2
55851	PSENEN	7.77	7.90	0.13	1.09	0.04	0.11	10.71	10.84	0.13	1.10	0.02	0.12	presenilin enhancer 2 homolog (C. elegans)
199223	TTC21A	6.50	6.43	-0.07	0.95	0.04	0.09	7.18	7.04	-0.15	0.90	0.00	0.03	tetratricopeptide repeat domain 21A
57732	ZFYVE28	6.86	6.79	-0.07	0.95	0.03	0.09	7.48	7.40	-0.08	0.95	0.04	0.12	zinc finger, FYVE domain containing 28
81029	WNT5B	6.72	6.64	-0.08	0.95	0.04	0.09	7.93	7.73	-0.20	0.87	0.02	0.07	wingless-type MMTV integration site family, member 5B
63898	SH2D4A	7.06	6.98	-0.08	0.95	0.05	0.09	9.17	8.79	-0.38	0.77	0.04	0.12	SH2 domain containing 4A
91544	UBXN11	6.85	6.76	-0.08	0.94	0.04	0.09	7.63	7.55	-0.08	0.95	0.04	0.12	UBX domain protein 11
147	ADRA1B	7.11	7.03	-0.08	0.94	0.03	0.09	7.78	7.52	-0.26	0.84	0.03	0.10	adrenergic, alpha-1B-, receptor
9942	XYLB	7.01	6.93	-0.09	0.94	0.03	0.09	8.77	8.54	-0.23	0.86	0.00	0.03	xylulokinase homolog (H. influenzae)
5865	RAB3B	6.68	6.59	-0.09	0.94	0.02	0.06	9.36	8.66	-0.70	0.62	0.01	0.07	RAB3B, member RAS oncogene family
56120	PCDHGB8P	6.80	6.71	-0.09	0.94	0.04	0.09	7.48	7.29	-0.18	0.88	0.01	0.06	protocadherin gamma subfamily B, 8 pseudogene
58538	MPP4	6.49	6.39	-0.10	0.93	0.02	0.06	6.95	6.78	-0.18	0.89	0.03	0.10	membrane protein, palmitoylated 4 (MAGUK p55 subfamily member 4)
114784	CSMD2	6.77	6.67	-0.10	0.93	0.01	0.06	7.70	7.43	-0.27	0.83	0.04	0.12	CUB and Sushi multiple domains 2
79413	ZBED2	6.71	6.61	-0.10	0.93	0.05	0.09	6.95	6.85	-0.10	0.93	0.01	0.05	zinc finger, BED-type containing 2
199221	DZIP1L	6.77	6.67	-0.10	0.93	0.01	0.06	7.26	7.18	-0.08	0.94	0.02	0.07	DAZ interacting protein 1-like
26266	SLC13A4	6.91	6.81	-0.11	0.93	0.03	0.09	7.42	7.30	-0.12	0.92	0.02	0.08	solute carrier family 13 (sodium/sulfate symporters), member 4
100303749	LOC100303749	6.23	6.12	-0.11	0.93	0.04	0.09	6.52	6.37	-0.15	0.90	0.01	0.06	LSM3 homolog, U6 small nuclear RNA associated (S. cerevisiae) pseudogene
435	ASL	7.21	7.10	-0.12	0.92	0.00	0.06	8.93	8.67	-0.27	0.83	0.03	0.10	argininosuccinate lyase
388948	LOC388948	6.53	6.41	-0.12	0.92	0.04	0.09	7.06	6.89	-0.18	0.88	0.01	0.05	hypothetical LOC388948
10505	SEMA4F	6.89	6.77	-0.12	0.92	0.01	0.06	8.18	7.98	-0.20	0.87	0.02	0.07	sema domain, immunoglobulin domain (Ig), transmembrane domain (TM) and short cytoplasmic domain, (semaphorin) 4F
28956	LAMTOR2	7.40	7.27	-0.13	0.92	0.02	0.06	8.58	8.44	-0.14	0.91	0.03	0.10	late endosomal/lysosomal adaptor, MAPK and MTOR activator 2
284344	LOC284344	6.41	6.27	-0.13	0.91	0.00	0.06	6.69	6.47	-0.22	0.86	0.01	0.04	hypothetical LOC284344
2817	GPC1	8.18	8.03	-0.14	0.90	0.03	0.06	9.91	9.60	-0.31	0.81	0.04	0.12	glypican 1
388610	TRNP1	7.29	7.12	-0.17	0.89	0.00	0.06	8.57	8.34	-0.24	0.85	0.05	0.14	TMF1-regulated nuclear protein 1
5675	PSG6	6.83	6.62	-0.21	0.86	0.02	0.06	8.18	7.65	-0.52	0.70	0.01	0.06	pregnancy specific beta-1-glycoprotein 6
51334	PRR16	7.40	7.16	-0.24	0.85	0.02	0.06	8.98	8.70	-0.28	0.83	0.03	0.10	proline rich 16
5339	PLEC	8.59	8.33	-0.26	0.84	0.04	0.09	9.90	9.71	-0.19	0.88	0.04	0.12	plectin
10370	CITED2	8.29	8.01	-0.28	0.83	0.01	0.06	10.21	9.89	-0.32	0.80	0.02	0.07	Cbp/p300-interacting transactivator, with Glu/Asp-rich carboxy-terminal domain, 2
7170	TPM3	10.13	9.80	-0.32	0.80	0.04	0.09	8.46	8.31	-0.16	0.90	0.01	0.07	tropomyosin 3
4633	MYL2	13.39	12.79	-0.61	0.66	0.01	0.06	6.65	6.56	-0.09	0.94	0.03	0.12	myosin, light chain 2, regulatory, cardiac, slow
